# Feasibility of social media health education plus exercise in older adults with possible sarcopenia

**DOI:** 10.1038/s41746-026-02585-7

**Published:** 2026-04-08

**Authors:** Ya Shi, Emma Stanmore, Lisa McGarrigle, Xiuhua Wang, Can Gu, Ying Ye, Chris Todd

**Affiliations:** 1 Xiangya School of Nursing, Central South University, Changsha, China; 2 School of Health Sciences, Faculty of Biology, Medicine & Health, University of Manchester, Manchester, UK; 3 School of Health Sciences and Manchester Academic Health Science Centre (MAH SC), University of Manchester, Manchester, UK; 4 Manchester University NHS Foundation Trust, Manchester, UK

**Keywords:** Health care, Medical research

## Abstract

Possible sarcopenia is prevalent among community-dwelling young-old adults, yet scalable, evidence-based social media interventions for prevention remain limited. This study evaluated the feasibility and acceptability of a Social media–based Health Education plus Exercise Programme (SHEEP) delivered via TikTok, along with its associated trial procedures. A single-arm mixed-methods feasibility study was conducted with 35 adults aged 60–69 years with possible sarcopenia in China. Participants received one week of health education followed by six weeks of home-based exercise training, with a six-week follow-up. Recruitment reached 38.5% of interested individuals, and 81.4% of eligible participants enroled. Adherence was high across intervention components, with most participants completing follow-up assessments. No exercise-related adverse events were reported, and both the intervention and study procedures were rated as highly acceptable. Exploratory analyses suggested improvements in muscle function, sarcopenia-related knowledge, self-efficacy, and exercise adherence. Qualitative findings indicated perceived physical and mental benefits and satisfaction with the programme. Overall, the SHEEP intervention and trial procedures were feasible, safe, and well accepted, meeting all pre-defined progression criteria. These findings support progression to a fully powered randomised controlled trial, with future studies optimising recruitment strategies and exploring hybrid delivery to enhance accessibility.

## Introduction

The 2024 conceptual definition established by the Global Leadership Initiative in Sarcopenia (GLIS) defines sarcopenia as an age-related generalised disease of skeletal muscle, marked by reduced muscle mass and strength, which may lead to compromised physical performance^1^. Sarcopenia can be classified into three categories (possible/probable, confirmed, and severe sarcopenia) according to distinct diagnostic criteria recommended by the European Working Group on Sarcopenia in Older People 2 (EWGSOP2)^2^ and the Asian Working Group for Sarcopenia 2 (AWGS2)^3^, aimed at addressing the requirements of the community and clinical practice more effectively.

Possible sarcopenia, characterised by diminished muscle strength with or without reduced physical performance^2,3^, is highly prevalent among community-dwelling older adults globally. Reported prevalence rates vary widely across populations, commonly ranging from 11% to over 45%^4–19^, and detailed country-specific data are provided in Supplementary Tables 1 and 2. When defined by the EWGSOP2 criteria, the prevalence of possible sarcopenia consistently exceeds that of confirmed and severe sarcopenia in community settings^12–16^. Although this pattern is somewhat less uniform when applying the AWGS2 criteria^17–19^, the overall evidence indicates that possible sarcopenia is generally more common than confirmed or severe sarcopenia among community-dwelling older adults in most studied populations.

In line with global advocacy for sarcopenia prevention in primary care^2,3^, research on interventions for possible sarcopenia has increased in recent years. For example, studies have demonstrated benefits from various approaches, including dual-task exercise (improving handgrip strength, gait speed and physical performance)^20^, protein supplementation (enhancing upper body physical function)^21^, combined resistance training and nutrition (increasing muscle mass and strength)^22^, and resistance band training (improving muscle mass and balance)^23^. However, in contrast to the studies on sarcopenia treatment, the research into sarcopenia prevention is rather under-researched. Our scoping review compared 59 studies about non-pharmacological interventions for community-dwelling older adults with three categories of sarcopenia and indicated that the number of studies involving older adults with sarcopenia (72.9%) far surpassed that of studies involving those with possible sarcopenia (11.9%)^24^. Among them, older people aged 60–69 years (18.5%) and 80–90 years (16.7%) have been studied less than aged 70–79 years (64.8%)^24^.

This gap is even more pronounced within digital health. While digital health interventions have gained attention in sarcopenia research^25–28^, fuelled by technological advances and the pandemic^29^, their integration into primary care is still emerging. Our recent systematic review identified sixteen studies about digital health exercise interventions in older adults with three categories of sarcopenia, but these concentrated primarily on the treatment of sarcopenia rather than its prevention, and no research explored the use of social media in sarcopenia prevention^30^. Besides, the corresponding meta-analysis indicated that standalone digital health exercise interventions, overall, showed no significant benefit on improving appendicular skeletal muscle mass index (MD = 0.16 kg/m², 95% CI: −0.03 to 0.36) or physical performance measures like the timed up-and-go test (SMD = −0.02, 95% CI: −0.40 to 0.37) in older people with sarcopenia, with limited data on handgrip strength^30^. Notably, subgroup analyses from the same meta-analysis suggested that combined interventions might be more promising^30^.

Hence, this study explored the feasibility of a multicomponent, social media-based intervention for community-dwelling older adults with possible sarcopenia. This work was informed by a prior development study, which used co-design with stakeholders to establish a theoretical framework and create the specific intervention: the Social media-based Health Education plus Exercise Programme (SHEEP), delivered via TikTok^31,32^. TikTok has become the predominant short-video platform for older Chinese adults, holding a 49.3% market share in this demographic^33^. Among its older users, those aged 60–65 and 66–70 years represent the largest cohorts (53.5% and 37.5%, respectively)^33^. Research indicates that during the COVID-19 pandemic, TikTok played a significant role in providing older adults with accessible health information, leading to positive health behaviour modifications^34–37^. Furthermore, the platform’s simple video-editing tools and format cater to the informational and practical needs of older users^38,39^. Given the absence of social media interventions for sarcopenia prevention, TikTok was identified as a culturally relevant and widely adopted medium to deliver a short-video-based intervention strategy to the target population in China. To our knowledge, SHEEP represents the first social media-based intervention designed specifically for sarcopenia prevention in community-dwelling older adults, addressing a critical gap in digital preventive health strategies.

Therefore, this study extends our prior research to further validate the feasibility and acceptability of the research design and the SHEEP intervention among young-old adults with possible sarcopenia living in the community. Different scholars have proposed varying definitions of ‘young-old’, commonly ranging from 60–69 to 65–74 years^40,41^. Given the limited evidence focusing on adults aged 60–69 years, as identified in our scoping review^24^, and considering that individuals aged 60 years and older are commonly defined as older adults in China^42^, this study defined young-old as those aged 60–69 years. The specific objectives include evaluating outcomes related to feasibility, including recruitment capability, data collection procedure, intervention suitability, outcome assessment process, researcher management ability, implementation feasibility, and research acceptability. The findings of this feasibility study will facilitate and establish a foundation for our forthcoming randomised controlled trial, and aid in supporting, guiding, and enhancing subsequent pertinent studies.

## Results

### Participant flow through the study

**Fig. 1:**
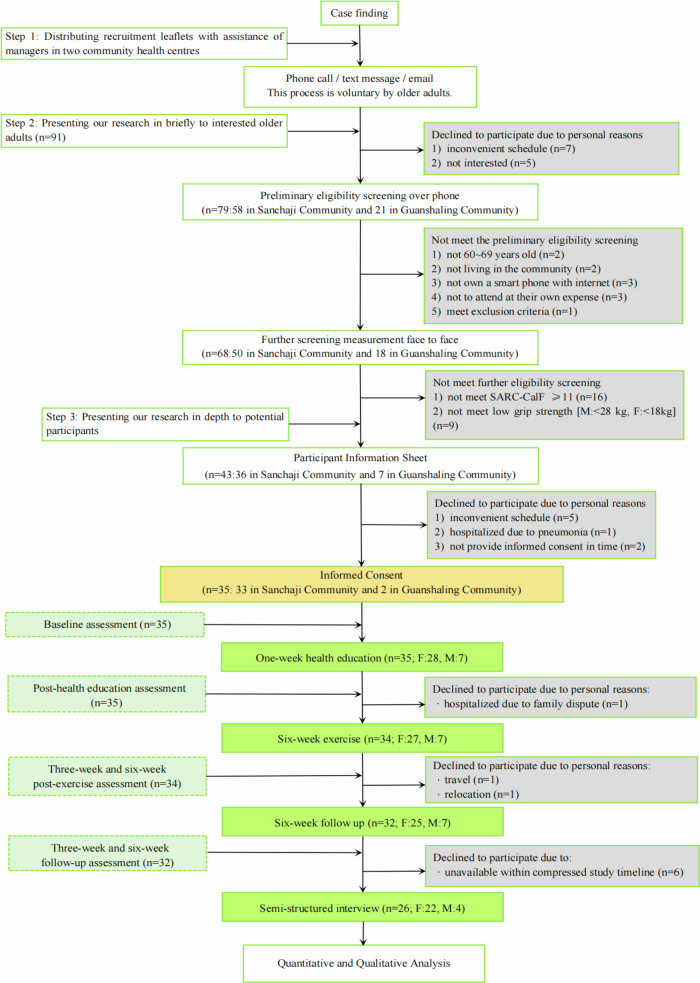
CONSORT flow diagram of the feasibility study. This diagram outlines participant recruitment, eligibility assessment, enrolment, intervention process, follow-up, and analysis in the SHEEP feasibility study.

Figure 1 presents the CONSORT flow diagram for this single-arm feasibility study, detailing participant progression to enhance transparency. The diagram illustrates the key stages: enrolment, allocation, intervention, follow-up and post-intervention interviews, and final analysis. It visually summarizes screening outcomes, reasons for exclusion, participant retention throughout the study phases, and the number of participants included in each analysis set.

### Primary outcomes

#### Recruitment capability

The information leaflets were distributed to older people who visited the community health centres, and the electronic leaflets were also sent to community WeChat groups. Between 06 May 2024 and 19 May 2024, in total 91 older adults expressed interest in joining this research programme through phone calls or text messages: 63 in Sanchaji Community Health Service Centre and 28 in Guanshaling Community Health Service Centre. These people learned of this research via paper information leaflets (*n* = 33, 36.3%), electronic leaflets in WeChat groups (*n* = 46, 50.5%), and word-of-mouth among residents (*n* = 12, 13.2%).

After a brief introduction to the study over the phone, 79 older adults accepted the preliminary eligibility screening over the phone (e.g. confirmed items 1–3 and 6 on eligibility list, and exclusion criteria); sixty-eight older people participated in further screening measurements, including SARC-CalF and handgrip strength according to AWGS 2^3^, face to face in the community, and 43 of them met all the screening criteria. After being given a full explanation of the study and receiving the Participant Information Sheet for the research, 35 participants provided informed consent and decided to join in this study: 33 in Sanchaji Community and 2 in Guanshaling Community, as shown in Fig. 1.

Two recruitment metrics were calculated: (1) The overall recruitment yield from all individuals who expressed initial interest was 38.5% (35/91); (2) The enrolment rate among screened-eligible individuals was 81.4% (35/43), indicating high acceptance of the study procedures among the target population. A marked difference was observed between the two communities: the enrolment rate among eligible individuals was 91.7% (33/36) in the Sanchaji Community compared to 28.6% (2/7) in the Guanshaling Community.

#### Participant characteristics

A total of 35 participants were included in the study. The mean age was 66.40 ± 2.90 years, with 80% aged 65–69 years and 20% aged 60–64 years. The majority of participants were female (80%). Educational attainment was distributed nearly evenly across three levels: primary school (31.4%), junior high school (34.3%), and senior high school (34.3%). Hypertension was the most common chronic condition (45.7%), followed by diabetes (22.9%), hyperlipidaemia (17.1%), lumbar disc herniation (5.7%), coronary heart disease (2.9%), and arthritis (2.9%). More than half of the participants (51.4%) reported taking medications for chronic diseases. Only one participant (2.9%) used a walking aid during the study period. Physical function and body composition measures included handgrip strength (right: 16.82 ± 4.90 kg; left: 15.86 ± 4.65 kg), 4-meter walking speed (1.29 ± 0.17 m/s), sit-to-stand time (8.13 ± 1.55 s), and body mass index (23.66 ± 2.70 kg/m²). No participants reported health problems significantly affecting their ability to perform physical activity. Detailed baseline characteristics of the total sample are presented in Table 1.

**Table 1:** Sample characteristics at baseline (*n* = 35)

Variable	M ± SD	Range	*N* (%)
Age, yrs	66.40 ± 2.90	60–69	
*Age group*			
60–64			7 (20.0)
65–69			28 (80.0)
Female			28 (80.0)
*Education*			
Primary school			11 (31.4)
Junior high school			12 (34.3)
Senior high school			12 (34.3)
*Long-term condition*			
Hypertension			16 (45.7)
Diabetes			8 (22.9)
Hyperlipidaemia			6 (17.1)
Coronary heart disease			1 (2.9)
Lumbar disc herniation			2 (5.7)
Arthritis			1 (2.9)
*Medication*			
Yes			18 (51.4)
*Significant health problems affecting the ability to undertake physical activity*			
Yes			0 (0.0)
*Mobility aid*			
Yes			1 (2.9)
*Physical function and body composition*			
Right handgrip strength, kg	16.82 ± 4.90	7.87–27.70	
Left handgrip strength, kg	15.86 ± 4.65	8.00–27.40	
4-metre walking speed, m/s	1.29 ± 0.17	0.73–1.70	
Sit to stand time, s	8.13 ± 1.55	5.73–4.00	
Body mass index, kg/m^2^	23.66 ± 2.70	18.36–29.47	

Baseline characteristics were also compared across participants grouped by study completion status [completers (*n* = 26), those completing follow-up without interview (*n* = 6), and early withdrawals (*n* = 3)]. No significant differences were found in any baseline variable between completers and those completing follow-up without interview, although statistical power was limited due to small withdrawal numbers. A full comparison is provided in Supplementary Note 1, Supplementary Tables 3 and 4.

#### Intervention acceptability

Of the 35 participants enroled, 32 (91.4% retention rate) completed the final follow-up assessment (T13). Among these 32 completers, 26 also participated in the semi-structured interview. The remaining nine participants did not complete the whole study process: three withdrew from the intervention and follow-up phase for external reasons (family emergency, travel, or relocation), and six who completed the final assessment were unavailable for the qualitative interview due to the compressed study timeline. Detailed participant flow is presented in Fig. 1. Process adherence was high across different stages: 100% during health education (viewing videos), 97.1% during the exercise intervention (attended $\ge$1 session), and 81.3% among follow-up completers who participated in the qualitative interview. No exercise-related adverse events (e.g., musculoskeletal injuries, falls, or cardiovascular incidents) were reported throughout the intervention and follow-up periods.

#### Acceptability of data collection

The duration required to collect secondary outcomes was recorded across five assessment timepoints (*n* = 32), ranging from 28.02 to 42.02 minutes in total. A repeated-measures ANOVA with Greenhouse–Geisser correction revealed no significant effect of timepoint on measurement duration [*F* (1.551, 48.078) = 1.090, *p* = 0.330)]. Mean durations per timepoint are detailed in Table 2.

**Table 2:** Measurement duration of secondary outcomes across five assessment timepoints (*n* = 32)

Assessment timepoint	Measurement time (mins) M ± SD (95%CIs)	*F*	*p*
T_0_	36.21 ± 2.59 (35.28, 37.14)	1.090	0.330
T_4_	36.35 ± 2.81 (35.34, 37.36)		
T_7_	35.94 ± 2.74 (34.95, 36.93)		
T_10_	35.55 ± 2.58 (34.62, 36.48)		
T_13_	35.37 ± 2.50 (34.47, 36.27)		

*T*_0_ Baseline, *T*_4_ Week 4 (exercise), *T*_7_ Week 7 (exercise), *T*_10_ Week 10 (follow-up), *T*_13_ Week 13 (follow-up). Degrees of freedom (Greenhouse–Geisser corrected): df₁ = 1.551, df₂ = 48.078.

#### Management ability

**Fig. 2:**
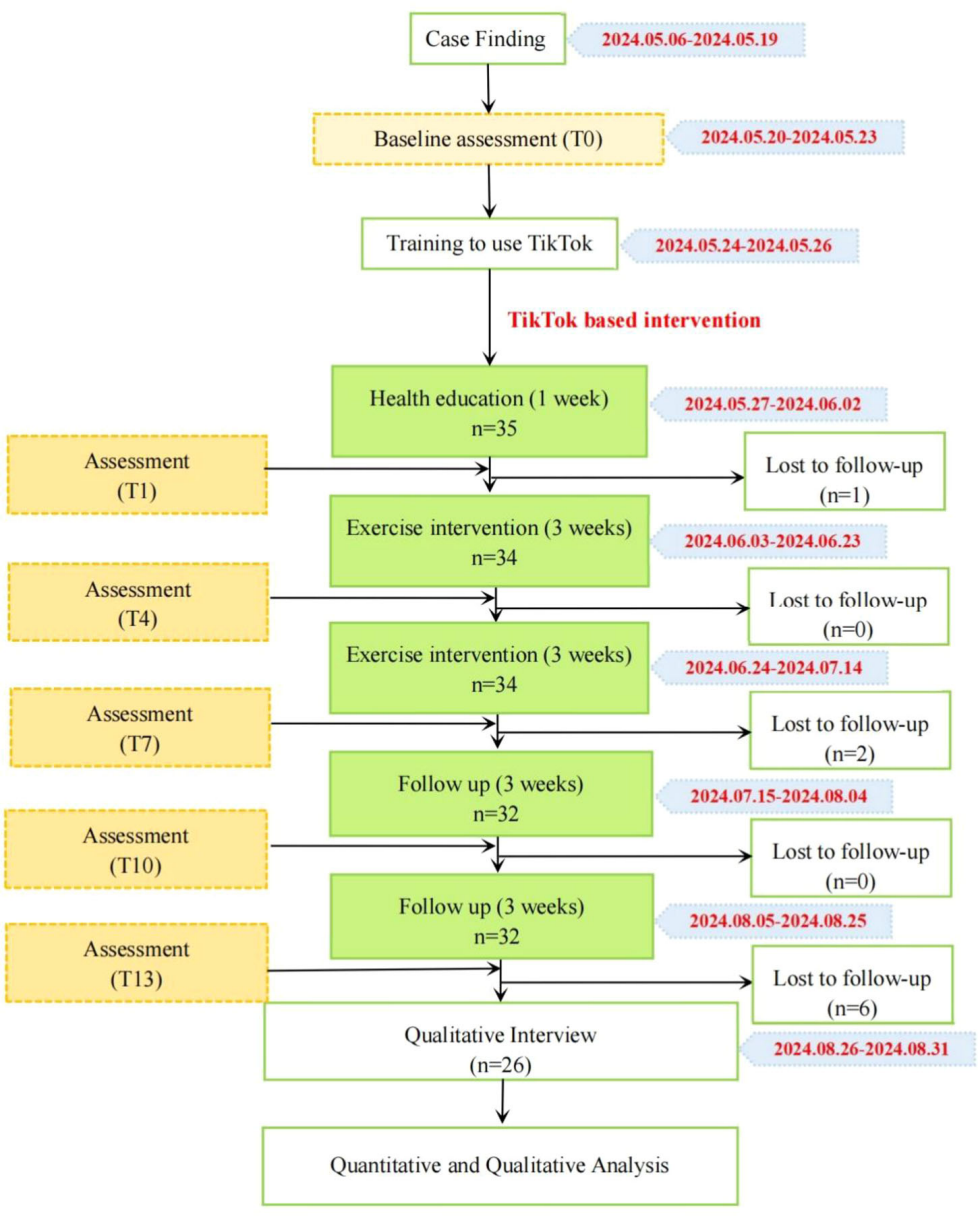
Timeline of the feasibility study procedures. The figure presents the sequence and duration of recruitment, baseline assessment, health education delivery, exercise training, follow-up assessment, and qualitative interviews.

The feasibility study was completed within the overall timeframe (17 weeks) of the original proposal, but its execution required significant schedule adjustments that yielded important management insights (Fig. 2). Initially, the recruitment and baseline phase took three weeks rather than the planned two, due to slower-than-anticipated community enrolment. While the core intervention and follow-up phases proceeded as scheduled, a critical constraint emerged in the final stage. To comply with the ethics-mandated data collection deadline, and compounded by the earlier recruitment delay, the period allocated for conducting semi-structured interviews was substantially compressed. This unforeseen compression resulted in scheduling conflicts that prevented six participants who had completed the final follow-up assessment from participating in the interviews.

## Secondary outcomes

### Changes in functional capacity

Changes in functional capacity indicators from baseline to Week 13 are presented in Table 3. All measured parameters demonstrated directional improvements over the study period. (1) Muscle strength: Both right and left handgrip strength increased progressively. For the total group, changes were statistically significant (both *p* < 0.001) with large effect sizes (Cohen’s *d* = +1.808 and +1.681, respectively). Similar increasing trends were observed in gender subgroups (all *p* ≤ 0.003). (2) Mobility: The 4-meter walking speed increased significantly (*p* < 0.001; Cohen’s *d* = +2.570), while the sit-to-stand time decreased (*p* < 0.001; Cohen’s *d* = –1.885), indicating improved lower-limb function and walking speed. These improvements were consistent across genders (all *p* ≤ 0.003). (3) Visual trends: The progression of all four functional outcomes across assessment timepoints is illustrated in Supplementary Fig. 1. The plots show consistent directional changes: a near-linear increase in handgrip strength, an upward trend in walking speed, and a downward trend in sit-to-stand time throughout the 13-week period. Gender-subgroup plots reflected similar patterns, suggesting a broadly consistent response to the intervention.

**Table 3:** Changes of functional capacity indicators from baseline to week 13

Variables	Assessment timepoint	M ± SD (95%CIs)		
		Total (n = 32)	Male (n = 7)	Female (n = 25)
Right handgrip strength, kg	T_0_	17.34 ± 4.73 (15.63, 19.04)	25.39 ± 2.02 (23.51, 27.26)	15.08 ± 1.92 (14.29, 15.87)
	T_4_	18.35 ± 4.77 (16.63, 20.07)	26.55 ± 1.97 (24.73, 28.38)	16.05 ± 1.80 (15.31, 16.79)
	T_7_	19.35 ± 4.85 (17.60, 21.10)	27.60 ± 2.27 (25.50, 29.69)	17.04 ± 1.93 (16.24, 17.84)
	T_10_	20.25 ± 4.93 (18.47, 22.03)	28.35 ± 2.75 (25.80, 30.89)	17.98 ± 2.25 (17.05, 18.91)
	T_13_	21.54 ± 5.28 (19.63, 23.44)	29.57 ± 3.79 (26.06, 33.07)	19.29 ± 2.89 (18.10, 20.48)
Change (T_13_ – T_0_)				
MD ± SD (95%CI)		4.20 ± 2.32 (3.36, 5.04)	4.18 ± 2.26 (2.09, 6.27)	4.21 ± 2.39 (3.22, 5.19)
Cohen’s *d* (95%CI)		+1.808 ( + 1.236, +2.369)	+1.849 ( + 0.566, +3.087)	+1.763 ( + 1.123, +2.387)
*t*		10.228	4.893	8.813
*p*		<0.001	0.003	<0.001
Left handgrip strength, kg	T_0_	16.08 ± 4.73 (14.37, 17.79)	23.36 ± 3.35 (20.27, 26.46)	14.04 ± 2.52 (13.00, 15.08)
	T_4_	17.08 ± 4.89 (15.32, 18.84)	24.82 ± 3.50 (21.59, 28.05)	14.91 ± 2.32 (13.95, 15.87)
	T_7_	18.06 ± 4.98 (16.27, 19.86)	26.04 ± 3.12 (23.16, 28.93)	15.83 ± 2.40 (14.84, 16.82)
	T_10_	19.08 ± 4.86 (17.33, 20.84)	26.89 ± 3.10 (24.02, 29.75)	16.90 ± 2.31 (15.95, 17.85)
	T_13_	19.95 ± 5.37 (18.01, 21.88)	28.14 ± 4.13 (24.32, 31.96)	17.66 ± 2.82 (16.49, 18.82)
Change (T_13_ – T_0_)				
MD ± SD (95%CI)		3.87 ± 2.30 (3.04, 4.70)	4.78 ± 1.88 (3.03, 6.52)	3.62 ± 2.38 (2.63, 4.60)
Cohen’s *d* (95%CI)		+1.681 ( + 1.134, +2.217)	+2.539 ( + 0.940, +4.104)	+1.521 ( + 0.933, +2.094)
*t*		9.510	6.717	7.605
*p*		<0.001	0.001	<0.001
4-metre walking speed, m/s	T_0_	1.29 ± 0.16 (1.23, 1.35)	1.24 ± 0.30 (0.97, 1.53)	1.30 ± 0.11 (1.25, 1.34)
	T_4_	1.32 ± 0.16 (1.26, 1.38)	1.26 ± 0.28 (0.99, 1.52)	1.33 ± 0.11 (1.29, 1.38)
	T_7_	1.35 ± 0.17 (1.29, 1.41)	1.28 ± 0.29 (1.02, 1.55)	1.37 ± 0.12 (1.33, 1.42)
	T_10_	1.39 ± 0.17 (1.33, 1.45)	1.31 ± 0.30 (1.03, 1.58)	1.41 ± 0.12 (1.37, 1.46)
	T_13_	1.44 ± 0.18 (1.38, 1.51)	1.35 ± 0.31 (1.06, 1.64)	1.47 ± 0.11 (1.42, 1.51)
Change (T_13_ – T_0_)				
MD ± SD (95%CI)		0.16 ± 0.06 (0.13, 0.18)	0.10 ± 0.06 (0.05, 0.15)	0.17 ± 0.05 (0.15, 0.19)
Cohen’s *d* (95%CI)		+2.570 ( + 1.840, +3.290)	+1.779 ( + 0.527, +2.986)	+3.239 ( + 2.242, +4.224)
*t*		14.538	4.708	16.193
*p*		<0.001	0.003	<0.001
Sit to stand time, s	T_0_	8.13 ± 1.51 (7.58, 8.67)	8.88 ± 2.41 (6.65, 11.11)	7.91 ± 1.13 (7.45, 8.38)
	T_4_	7.80 ± 1.47 (7.27, 8.33)	8.63 ± 2.51 (6.30, 10.95)	7.57 ± 0.98 (7.16, 7.97)
	T_7_	7.49 ± 1.48 (6.96, 8.02)	8.43 ± 2.58 (6.05, 10.82)	7.22 ± 0.91 (6.85, 7.60)
	T_10_	7.14 ± 1.48 (6.61, 7.68)	8.22 ± 2.54 (5.87, 10.57)	6.84 ± 0.89 (6.47, 7.20)
	T_13_	6.85 ± 1.53 (6.30, 7.41)	8.00 ± 2.66 (5.54, 10.46)	6.53 ± 0.89 (6.17, 6.90)
Change (T_13_ – T_0_)				
MD ± SD (95%CI)		−1.27 ± 0.67 (−1.51, −1.03)	−0.88 ± 0.39 (−1.24, −0.52)	−1.38 ± 0.70 (−1.67, −1.09)
Cohen’s *d* (95%CI)		−1.885 (−2.461, −1.298)	−2.271 (−3.706, −0.798)	−1.968 (−2.639, −1.283)
*t*		−10.664	−6.009	−9.839
*p*		<0.001	0.001	<0.001

*T*_0_ Baseline, *T*_*1*_ Week 1 (health education), *T*_4_ Week 4 (exercise), *T*_7_ Week 7 (exercise), *T*_10_ Week 10 (follow-up), *T*_13_ Week 13 (follow-up). MD mean difference from baseline to week 13. The symbol ‘+’ denotes a positive direction of intervention effect, whereas the symbol ‘-’ indicates a negative direction of intervention effect. All p-values are unadjusted for multiple comparisons and should be interpreted as exploratory.

#### Changes in body composition

Changes in body composition from baseline to Week 13 are summarized in Table 4. The results presented a mixed pattern, with stability in some measures and change in others. (1) Regional muscle mass: Total skeletal muscle mass and skeletal muscle index remained stable in the total group (both *p* > 0.64). In contrast, trunk and upper-extremity skeletal muscle mass showed significant increases (all *p* ≤ 0.003; Cohen’s *d* range: +0.58 to +0.70), particularly among female participants. Lower-extremity skeletal muscle mass did not demonstrate a consistent positive change. (2) Body weight, adiposity, and dimensions: Body weight and BMI increased significantly (both *p* < 0.001; Cohen’s *d* ≈ +1.0). Total body fat also increased (*p* = 0.012), with a notable rise in male participants (*p* = 0.027; Cohen’s *d* = +1.47). Concurrently, upper arm dimensions (muscle and total) increased (both *p* < 0.001), while abdominal circumference showed a non-significant decreasing trend (*p* = 0.161) and calf circumferences remained stable. (3) Visual trends: The progression of key body composition indicators is illustrated in Supplementary Fig. 2. The plots corroborate the statistical findings, showing upward trends for weight, BMI, and upper arm dimensions, a gradual increase in trunk and upper-extremity muscle mass, and stable trajectories for total muscle mass and lower-extremity measures.

**Table 4:** Changes of body composition indicators from baseline to week 13

Variables	Assessment timepoint	M ± SD (95%CIs)		
		Total (*n* = 32)	Male (*n* = 7)	Female (*n* = 25)
Skeletal muscle mass, kg	T_0_	24.53 ± 5.38 (22.59, 26.47)	32.33 ± 5.39(27.34, 37.32)	22.35 ± 2.73(21.22, 23.48)
	T_4_	24.72 ± 5.28 (22.82, 26.62)	32.50 ± 4.64(28.21, 36.79)	22.54 ± 2.82(21.37, 23.71)
	T_7_	24.62 ± 5.23 (22.73, 26.50)	31.91 ± 5.03(27.26, 36.57)	22.58 ± 3.02(21.33, 23.82)
	T_10_	24.61 ± 5.12 (22.77, 26.46)	32.19 ± 4.20(28.30, 36.07)	22.49 ± 2.84(21.32, 23.66)
	T_13_	24.62 ± 5.02 (22.81, 26.43)	31.54 ± 4.96(26.95, 36.13)	22.68 ± 2.92(21.48, 23.89)
Change (T_13_ – T_0_)				
MD ± SD (95%CI)		0.09 ± 1.11 (−0.31, 0.49)	−0.79 ± 1.25 (−1.94, 0.37)	0.34 ± 0.96 (−0.06, 0.73)
Cohen’s *d* (95%CI)		+0.081 (−0.266, +0.428)	−0.628 (-1.426, +0.211)	+0.350 (−0.058, +0.751)
*t/Z*		0.461	−1.355^a^	1.750
*p*		0.648	0.176	0.093
Skeletal mass index, kg/m^2^	T_0_	7.95 ± 1.38 (7.45, 8.45)	9.96 ± 1.35(8.71, 11.21)	7.39 ± 0.72(7.10, 7.69)
	T_4_	7.96 ± 1.32 (7.49, 8.43)	9.76 ± 1.16(8.69, 10.83)	7.46 ± 0.83(7.11, 7.80)
	T_7_	7.95 ± 1.26 (7.50, 8.41)	9.56 ± 1.31(8.35, 10.77)	7.50 ± 0.82(7.17, 7.84)
	T_10_	7.95 ± 1.29 (7.48, 8.42)	9.71 ± 1.12(8.68, 10.75)	7.46 ± 0.82(7.12, 7.80)
	T_13_	7.95 ± 1.31 (7.48, 8.42)	9.63 ± 1.40(8.33, 10.93)	7.48 ± 0.81(7.14, 7.81)
Change (T_13_ – T_0_)				
MD ± SD (95%CI)		−0.01 ± 0.34 (−0.13, 0.12)	−0.33 ± 0.40 (−0.70, 0.04)	0.08 ± 0.27 (−0.03, 0.19)
Cohen’s *d* (95%CI)		−0.018 (−0.365, +0.328)	−0.815 (-1.659, +0.075)	+0.314 (−0.091, +0.713)
*t*		−0.103	-2.157	1.572
*p*		0.918	0.074	0.129
Trunk skeletal muscle mass, kg	T_0_	15.88 ± 2.52 (14.97, 16.79)	19.36 ± 2.03(17.48, 21.24)	14.91 ± 1.64(14.23, 15.58)
	T_4_	16.04 ± 2.66 (15.09, 17.00)	19.77 ± 2.26(17.68, 21.86)	15.00 ± 1.63(14.33, 15.67)
	T_7_	16.01 ± 2.56 (15.09, 16.93)	19.59 ± 2.03(17.71, 21.47)	15.01 ± 1.62(14.34, 15.68)
	T_10_	16.18 ± 2.66 (15.23, 17.14)	20.19 ± 1.75(18.57, 21.80)	15.06 ± 1.54(14.43, 15.70)
	T_13_	16.33 ± 2.56 (15.41, 17.25)	19.87 ± 1.93(18.09, 21.66)	15.34 ± 1.68(14.65, 16.03)
Change (T_13_ – T_0_)				
MD ± SD (95%CI)		0.45 ± 0.64 (0.22, 0.68)	0.51 ± 0.92 (−0.34, 1.36)	0.43 ± 0.57 (0.20, 0.67)
Cohen’s *d* (95%CI)		+0.700 (+0.308, +1.083)	+0.560 (−0.262, +1.343)	+0.762 (+0.309, +1.203)
*t*		3.958	1.480	3.810
*p*		<0.001	0.189	0.001
Right upper-extremity skeletal muscle mass, kg	T_0_	1.69 ± 0.42 (1.54, 1.84)	2.26 ± 0.34(1.95, 2.58)	1.53 ± 0.27(1.42, 1.64)
	T_4_	1.71 ± 0.44 (1.56, 1.87)	2.34 ± 0.39(1.98, 2.69)	1.54 ± 0.26(1.43, 1.64)
	T_7_	1.70 ± 0.41 (1.55, 1.85)	2.29 ± 0.37(1.95, 2.63)	1.54 ± 0.25(1.44, 1.64)
	T_10_	1.73 ± 0.43 (1.58, 1.89)	2.39 ± 0.31(2.11, 2.67)	1.55 ± 0.24(1.45, 1.65)
	T_13_	1.76 ± 0.42 (1.60, 1.91)	2.35 ± 0.34(2.03, 2.67)	1.59 ± 0.27(1.48, 1.70)
Change (T_13_ – T_0_)				
MD ± SD (95%CI)		0.07 ± 0.11 (0.03, 0.11)	0.09 ± 0.13 (−0.04, 0.21)	0.07 ± 0.10 (0.02, 0.11)
Cohen’s *d* (95%CI)		+0.662 (+0.275, +1.041)	+0.649 (−0.195, +1.451)	+0.657 (+0.218, +1.085)
*t*		3.747	1.718	3.284
*p*		0.001	0.137	0.003
Left upper-extremity skeletal muscle mass, kg	T_0_	1.66 ± 0.41 (1.51, 1.80)	2.22 ± 0.35(1.90, 2.54)	1.50 ± 0.26(1.39, 1.61)
	T_4_	1.69 ± 0.44 (1.53, 1.84)	2.30 ± 0.40(1.93, 2.68)	1.51 ± 0.26(1.41, 1.62)
	T_7_	1.67 ± 0.42 (1.52, 1.82)	2.27 ± 0.36(1.93, 2.60)	1.51 ± 0.25(1.40, 1.61)
	T_10_	1.69 ± 0.44 (1.53, 1.85)	2.35 ± 0.33(2.05, 2.66)	1.50 ± 0.24(1.40, 1.60)
	T_13_	1.72 ± 0.42 (1.57, 1.87)	2.30 ± 0.34(1.99, 2.62)	1.56 ± 0.27(1.45, 1.67)
Change (T_13_ – T_0_)				
MD ± SD (95%CI)		0.06 ± 0.11 (0.02, 0.10)	0.08 ± 0.16 (−0.07, 0.23)	0.06 ± 0.09 (0.02, 0.10)
Cohen’s *d* (95%CI)		+0.581 (+0.202, +0.953)	+0.505 (−0.305, +1.279)	+0.623 (+0.188, +1.047)
*t*		3.289	1.336	3.114
*p*		0.003	0.230	0.005
Right lower-extremity skeletal muscle mass, kg	T_0_	8.22 ± 2.10 (7.46, 8.98)	11.45 ± 1.83(9.76, 13.15)	7.32 ± 0.98(6.91, 7.72)
	T_4_	8.25 ± 2.01 (7.53, 8.98)	11.19 ± 1.55(9.75, 12.62)	7.43 ± 1.19(6.94, 7.92)
	T_7_	8.31 ± 2.04 (7.57, 9.04)	11.07 ± 1.91(9.31, 12.84)	7.53 ± 1.26(7.01, 8.05)
	T_10_	8.32 ± 1.98 (7.61, 9.04)	11.14 ± 1.60(9.66, 12.62)	7.53 ± 1.20(7.04, 8.03)
	T_13_	8.21 ± 1.89 (7.53, 8.90)	10.77 ± 1.88(9.03, 12.50)	7.50 ± 1.15(7.02, 7.97)
Change (T_13_ – T_0_)				
MD ± SD (95%CI)		−0.01 ± 0.61 (−0.23, 0.21)	−0.68 ± 0.51 (−1.16, −0.21)	0.18 ± 0.49 (−0.02, 0.38)
Cohen’s *d* (95%CI)		−0.011 (−0.357, +0.336)	−1.334 (−2.352, −0.263)	+0.375 (−0.035, +0.777)
*t*		−0.061	−3.528	1.874
*p*		0.952	0.012	0.073
Left lower-extremity skeletal muscle mass, kg	T_0_	8.15 ± 1.98 (7.44, 8.87)	11.28 ± 1.39(10.00, 12.57)	7.28 ± 0.97(6.88, 7.68)
	T_4_	8.06 ± 1.93 (7.36, 8.75)	10.90 ± 1.29(9.71, 12.09)	7.26 ± 1.18(6.77, 7.74)
	T_7_	8.07 ± 1.83 (7.41, 8.73)	10.69 ± 1.56(9.25, 12.13)	7.34 ± 1.07(6.89, 7.78)
	T_10_	8.10 ± 1.80 (7.45, 8.74)	10.77 ± 1.19(9.67, 11.87)	7.35 ± 1.07(6.91, 7.79)
	T_13_	8.03 ± 1.77 (7.39, 8.67)	10.46 ± 1.67(8.92, 12.01)	7.35 ± 1.06(6.91, 7.78)
Change (T_13_ – T_0_)				
MD ± SD (95%CI)		−0.13 ± 0.61 (−0.34,0.09)	−0.82 ± 0.65 (−1.42, −0.22)	0.07 ± 0.44 (−0.11, 0.25)
Cohen’s *d* (95%CI)		−0.207 (−0.556, +0.145)	−1.266 (−2.259, −0.222)	+0.158 (−0.238, +0.551)
*t/Z*		−0.468^a^	−3.349	0.790
*p*		0.640	0.015	0.437
Upper arm dimension, cm	T_0_	25.89 ± 2.08 (25.14, 26.64)	26.43 ± 2.57(24.05, 28.81)	25.74 ± 1.95(24.93, 26.55)
	T_4_	26.34 ± 2.14 (25.57, 27.11)	27.50 ± 2.27(25.40, 29.61)	26.02 ± 2.03(25.18, 26.86)
	T_7_	26.31 ± 2.00 (25.59, 27.03)	27.61 ± 1.87(25.88, 29.33)	25.95 ± 1.92(25.16, 26.74)
	T_10_	26.57 ± 1.97 (25.86, 27.27)	28.00 ± 1.90(26.24, 29.76)	26.16 ± 1.82(25.41, 26.92)
	T_13_	26.70 ± 2.08 (25.95, 27.45)	28.16 ± 1.58(26.70, 29.62)	26.30 ± 2.04(25.45, 27.14)
Change (T_13_ – T_0_)				
MD ± SD (95%CI)		0.81 ± 1.16 (0.39, 1.23)	1.73 ± 1.67 (0.19, 3.27)	0.56 ± 0.86 (0.20, 0.91)
Cohen’s *d* (95%CI)		+0.699 (+0.307, +1.082)	+1.036 (+0.074, +1.947)	+0.648 (+0.210, +1.075)
*t/Z*		−3.581^a^	2.742	3.239
*p*		<0.001	0.034	0.003
Upper arm muscle dimension, cm	T_0_	20.86 ± 1.59 (20.28, 21.43)	22.33 ± 1.82(20.64, 24.01)	20.44 ± 1.27(19.92, 20.97)
	T_4_	21.10 ± 1.65 (20.51, 21.70)	22.89 ± 1.68(21.33, 24.44)	20.60 ± 1.27(20.08, 21.13)
	T_7_	21.09 ± 1.62 (20.51, 21.68)	22.89 ± 1.50(21.50, 24.27)	20.59 ± 1.28(20.06, 21.11)
	T_10_	21.15 ± 1.62 (20.57, 21.74)	23.23 ± 1.35(21.98, 24.47)	20.57 ± 1.15(20.10, 21.05)
	T_13_	21.29 ± 1.58 (20.72, 21.86)	23.19 ± 1.21(22.07, 24.30)	20.76 ± 1.23(20.26, 21.27)
Change (T_13_ – T_0_)				
MD ± SD (95%CI)		0.44 ± 0.66 (0.20, 0.68)	0.86 ± 1.19 (−0.25, 1.96)	0.32 ± 0.37 (0.17, 0.47)
Cohen’s *d* (95%CI)		+0.664 (+0.276, +1.043)	+0.718 (−0.145, +1.536)	+0.858 (+0.391, +1.312)
*t/Z*		-3.701^a^	1.899	4.289
*p*		<0.001	0.106	<0.001
Height, cm	T_0_	156.49 ± 5.83 (154.39, 158.59)	163.86 ± 2.84(161.23, 166.49)	154.43 ± 4.65(152.51, 156.35)
	T_4_	156.38 ± 5.81 (154.28, 158.47)	163.60 ± 2.85(160.96, 166.24)	154.35 ± 4.69(152.42, 156.29)
	T_7_	156.41 ± 5.82 (154.31, 158.51)	163.74 ± 2.75(161.20, 166.29)	154.36 ± 4.67(152.43, 156.29)
	T_10_	156.37 ± 5.84 (154.27, 158.48)	163.70 ± 2.64(161.26, 166.14)	154.32 ± 4.72(152.37, 156.27)
	T_13_	156.31 ± 5.85 (154.20, 158.42)	163.60 ± 2.67(161.13, 166.07)	154.27 ± 4.75(152.31, 156.23)
Change (T_13_ – T_0_)				
MD ± SD (95%CI)		−0.18 ± 0.26 (−0.27, −0.09)	−0.26 ± 0.25 (−0.49, −0.03)	−0.16 ± 0.26 (−0.26, −0.05)
Cohen’s *d* (95%CI)		−0.695 (−1.078, −0.304)	−1.026 (−1.933, −0.067)	−0.604 (−1.026, −0.171)
*t/Z*		−3.933	−2.388^a^	−3.019
*p*		<0.001	0.017	0.006
Weight, kg	T_0_	57.65 ± 9.07 (54.38, 60.92)	68.86 ± 8.40(61.09, 76.63)	54.51 ± 6.46(51.84, 57.18)
	T_4_	58.30 ± 9.05 (55.04, 61.56)	69.67 ± 8.72(61.61, 77.74)	55.12 ± 6.20(52.56, 57.68)
	T_7_	58.50 ± 8.96 (55.27, 61.73)	69.53 ± 8.77(61.42, 77.64)	55.41 ± 6.25(52.83, 57.99)
	T_10_	59.00 ± 8.90 (55.79, 62.21)	69.99 ± 8.36(62.25, 77.72)	55.93 ± 6.31(53.33, 58.53)
	T_13_	58.95 ± 8.90 (55.74, 62.16)	69.96 ± 8.47(62.12, 77.79)	55.87 ± 6.26(53.29, 58.46)
Change (T_13_ – T_0_)				
MD ± SD (95%CI)		1.30 ± 1.31 (0.83, 1.77)	1.10 ± 1.06 (0.12, 2.08)	1.36 ± 1.38 (0.79, 1.93)
Cohen’s *d* (95%CI)		+0.998 (+0.567, +1.419)	+1.036 (+0.075, +1.947)	+0.986 (+0.499, +1.459)
*t*		5.648	2.742	4.929
*p*		<0.001	0.034	<0.001
Body mass index, kg/m^2^	T_0_	23.45 ± 2.66 (22.49, 24.41)	25.63 ± 2.93(22.93, 28.34)	22.84 ± 2.28(21.90, 23.78)
	T_4_	23.76 ± 2.71 (22.78, 24.74)	26.02 ± 3.03(23.21, 28.82)	23.13 ± 2.29(22.18, 24.07)
	T_7_	23.83 ± 2.68 (22.86, 24.80)	25.92 ± 3.06(23.08, 28.75)	23.25 ± 2.31(22.29, 24.20)
	T_10_	24.04 ± 2.60 (23.11, 24.98)	26.10 ± 2.90(23.42, 28.79)	23.47 ± 2.24(22.54, 24.39)
	T_13_	24.05 ± 2.62 (23.10, 24.99)	26.12 ± 2.94(23.40, 28.85)	23.46 ± 2.26(22.53, 24.40)
Change (T_13_ – T_0_)				
MD ± SD (95%CI)		0.60 ± 0.57 (0.39, 0.80)	0.49 ± 0.36 (0.16, 0.82)	0.63 ± 0.62 (0.37, 0.88)
Cohen’s *d* (95%CI)		+1.052 (+0.613, +1.480)	+1.375 (+0.289, +2.410)	+1.017 (+0.524, +1.495)
*t*		5.950	3.638	5.083
*p*		<0.001	0.011	<0.001
Body fat, kg	T_0_	12.23 ± 3.86 (10.84, 13.62)	10.10 ± 1.86(8.38, 11.82)	12.82 ± 4.09(11.14, 14.51)
	T_4_	12.86 ± 3.77 (11.50, 14.22)	10.94 ± 1.72(9.35, 12.53)	13.39 ± 4.03(11.73, 15.06)
	T_7_	12.88 ± 4.08 (11.41, 14.36)	11.74 ± 2.21(9.70, 13.79)	13.20 ± 4.45(11.37, 15.04)
	T_10_	13.66 ± 3.75 (12.31, 15.01)	12.63 ± 2.09(10.70, 14.56)	13.95 ± 4.09(12.26, 15.64)
	T_13_	13.38 ± 4.51 (11.75, 15.00)	12.27 ± 1.90(10.52, 14.02)	13.68 ± 4.99(11.63, 15.74)
Change (T_13_ – T_0_)				
MD ± SD (95%CI)		1.15 ± 2.43 (0.27, 2.02)	2.17 ± 1.48 (0.80, 3.54)	0.86 ± 2.59 (−0.21, 1.93)
Cohen’s *d* (95%CI)		+0.471 (+0.102, +0.833)	+1.469 (+0.345, +2.542)	+0.332 (−0.074, +0.732)
*t/Z*		2.665	-2.205^a^	1.659
*p*		0.012	0.027	0.110
Body fat percentage, %	T_0_	21.45 ± 6.24 (19.20, 23.70)	14.81 ± 3.09(11.95, 17.67)	23.31 ± 5.60(21.00, 25.62)
	T_4_	22.27 ± 6.22 (20.03, 24.51)	15.86 ± 1.80(14.19, 17.52)	24.07 ± 5.81(21.67, 26.47)
	T_7_	22.21 ± 6.48 (19.87, 24.55)	17.10 ± 3.14(14.20, 20.00)	23.64 ± 6.49(20.96, 26.32)
	T_10_	23.25 ± 5.95 (21.11, 25.40)	18.03 ± 2.81(15.43, 20.63)	24.72 ± 5.79(22.33, 27.11)
	T_13_	22.71 ± 6.83 (20.24, 25.17)	17.39 ± 2.44(15.13, 19.65)	24.20 ± 6.94(21.33, 27.06)
Change (T_13_ – T_0_)				
MD ± SD (95%CI)		1.26 ± 3.65 (−0.06, 2.57)	2.57 ± 1.54 (1.15, 3.99)	0.89 ± 4.00 (−0.76, 2.54)
Cohen’s *d* (95%CI)		+0.344 (−0.015, +0.698)	+1.674 (+0.466, +2.834)	+0.222 (−0.177, +0.617)
*t/Z*		1.947	−2.366^a^	1.111
*p*		0.061	0.018	0.278
Protein, kg	T_0_	8.79 ± 1.77 (8.16, 9.43)	11.36 ± 1.75(9.74, 12.97)	8.08 ± 0.91(7.70, 8.45)
	T_4_	8.83 ± 1.73 (8.20, 9.45)	11.34 ± 1.50(9.95, 12.73)	8.12 ± 0.96(7.72, 8.52)
	T_7_	8.83 ± 1.73 (8.20, 9.45)	11.26 ± 1.65(9.73, 12.78)	8.14 ± 1.00(7.73, 8.56)
	T_10_	8.80 ± 1.70 (8.19, 9.42)	11.29 ± 1.40(9.99, 12.58)	8.11 ± 0.96(7.71, 8.51)
	T_13_	8.86 ± 1.68 (8.25, 9.46)	11.23 ± 1.54(9.80, 12.66)	8.19 ± 0.97(7.79, 8.59)
Change (T_13_ – T_0_)				
MD ± SD (95%CI)		0.06 ± 0.36 (−0.07, 0.19)	−0.13 ± 0.41 (−0.51, 0.25)	0.12 ± 0.33 (−0.02, 0.25)
Cohen’s *d* (95%CI)		+0.175 (−0.175, +0.523)	−0.316 (−1.065, +0.457)	+0.352 (−0.056, +0.752)
*t*		0.992	−0.836	1.758
*p*		0.329	0.435	0.092
Bone mineral, kg	T_0_	2.91 ± 0.66 (2.68, 3.15)	3.73 ± 0.86(2.94, 4.52)	2.69 ± 0.35(2.54, 2.83)
	T_4_	2.94 ± 0.65 (2.71, 3.18)	3.80 ± 0.71(3.14, 4.45)	2.70 ± 0.38(2.55, 2.86)
	T_7_	2.93 ± 0.63 (2.70, 3.16)	3.71 ± 0.75(3.02, 4.41)	2.71 ± 0.39(2.55, 2.87)
	T_10_	2.91 ± 0.61 (2.69, 3.13)	3.68 ± 0.66(3.07, 4.30)	2.69 ± 0.37(2.54, 2.84)
	T_13_	2.89 ± 0.62 (2.66, 3.11)	3.62 ± 0.78(2.90, 4.35)	2.68 ± 0.38(2.52, 2.84)
Change (T_13_ – T_0_)				
MD ± SD (95%CI)		−0.03 ± 0.17 (−0.09, 0.03)	−0.11 ± 0.19 (−0.28, 0.07)	−0.01 ± 0.16 (−0.07, 0.06)
Cohen’s *d* (95%CI)		−0.157 (−0.505, +0.193)	−0.562 (−1.346, +0.261)	−0.025 (−0.417, +0.367)
*t/Z*		−0.890	−1.016^a^	−0.126
*p*		0.380	0.310	0.901
Abdominal circumference, cm	T_0_	80.72 ± 8.63 (77.61, 83.83)	86.07 ± 10.28(76.57, 95.58)	79.22 ± 7.69(76.05, 82.39)
	T_4_	80.53 ± 8.80 (77.36, 83.70)	86.36 ± 11.26(75.95, 96.77)	78.90 ± 7.47(75.82, 81.98)
	T_7_	80.56 ± 8.83 (77.38, 83.75)	86.86 ± 10.77(76.89, 96.82)	78.80 ± 7.54(75.69, 81.91)
	T_10_	80.16 ± 9.06 (76.89, 83.42)	86.71 ± 11.97(75.65, 97.78)	78.32 ± 7.37(75.28, 81.36)
	T_13_	79.88 ± 9.06 (76.61, 83.14)	87.36 ± 11.24(76.96, 97.75)	77.78 ± 7.31(74.76, 80.80)
Change (T_13_ – T_0_)				
MD ± SD (95%CI)		−0.84 ± 3.32 (−2.04, 0.35)	1.29 ± 2.78 (−1.29, 3.86)	−1.44 ± 3.26 (−2.78, −0.10)
Cohen’s *d* (95%CI)		−0.254 (−0.604, +0.100)	+0.462 (−0.338, +1.229)	−0.442 (−0.849, −0.027)
*t*		−1.438	1.223	−2.210
*p*		0.161	0.267	0.037
Right calf circumference, cm	T_0_	32.23 ± 1.65 (31.64, 32.83)	32.93 ± 1.62(31.43, 34.43)	32.04 ± 1.63(31.37, 32.71)
	T_4_	32.20 ± 1.68 (31.60, 32.81)	32.93 ± 1.59(31.46, 34.40)	32.00 ± 1.68(31.31, 32.69)
	T_7_	32.16 ± 1.67 (31.55, 32.76)	32.93 ± 1.48(31.56, 34.30)	31.94 ± 1.69(31.24, 32.64)
	T_10_	32.17 ± 1.69 (31.56, 32.78)	33.00 ± 1.32(31.78, 34.22)	31.94 ± 1.73(31.23, 32.65)
	T_13_	32.09 ± 1.78 (31.45, 32.74)	32.86 ± 1.31(31.64, 34.07)	31.88 ± 1.86(31.11, 32.65)
Change (T_13_ – T_0_)				
MD ± SD (95%CI)		−0.14 ± 0.84 (−0.44, 0.16)	−0.07 ± 0.93 (−0.93, 0.79)	−0.16 ± 0.83 (−0.50, 0.18)
Cohen’s *d* (95%CI)		−0.168 (−0.516, +0.182)	−0.077 (−0.816, +0.668)	−0.194 (−0.588, +0.204)
*t/Z*		−1.050^a^	−0.203	−1.046^a^
*p*		0.294	0.846	0.296
Left calf circumference, cm	T_0_	32.47 ± 1.62 (31.88, 33.05)	33.21 ± 1.70(31.64, 34.79)	32.26 ± 1.57(31.61, 32.91)
	T_4_	32.41 ± 1.64 (31.81, 33.00)	33.14 ± 1.73(31.55, 34.74)	32.20 ± 1.59(31.54, 32.86)
	T_7_	32.38 ± 1.63 (31.79, 32.96)	33.00 ± 1.53(31.59, 34.41)	32.20 ± 1.65(31.52, 32.88)
	T_10_	32.34 ± 1.68 (31.74, 32.95)	33.21 ± 1.68(31.66, 34.77)	32.10 ± 1.63(31.43, 32.77)
	T_13_	32.28 ± 1.77 (31.64, 32.92)	33.21 ± 1.58(31.76, 34.67)	32.02 ± 1.76(31.29, 32.75)
Change (T_13_ – T_0_)				
MD ± SD (95%CI)		−0.19 ± 0.79 (−0.47, 0.10)	0.00 ± 0.82 (−0.76, 0.76)	−0.24 ± 0.79 (−0.57, 0.09)
Cohen’s *d* (95%CI)		−0.237 (−0.587, +0.116)	0.000 (−0.741, +0.741)	−0.303 (−0.701, +0.101)
*t/Z*		−1.100^a^	0.000	−1.301^a^
*p*		0.271	1.000	0.193

‘a’ means that difference between T13 and T0 did not meet normality assumption (Shapiro-Wilk test *p* < 0.05), thus Wilcoxon signed-rank test was used. *T*_0_ Baseline, *T*_1_ Week 1 (health education), *T*_4_ Week 4 (exercise), *T*_7_ Week 7 (exercise), *T*_10_ Week 10 (follow-up), *T*_13_ Week 13 (follow-up). *MD* mean difference from baseline to week 13. The symbol ‘+’ denotes a positive direction of intervention effect, whereas the symbol ‘-’ indicates a negative direction of intervention effect. All *p*-values are unadjusted for multiple comparisons and should be interpreted as exploratory.

#### Changes in knowledge, psychosocial factors, and behavioural indicators

Changes in knowledge, psychosocial, and behavioural indicators from baseline to Week 13 are presented in Table 5. Marked improvements were observed in knowledge, with notable shifts in several psychosocial and behavioural domains. (1) Knowledge and nutrition: Sarcopenia prevention knowledge scores increased substantially immediately after the initial health education session (T1) and remained elevated throughout the study. The change from baseline to Week 13 was highly significant (*p* < 0.001) with an exceptionally large effect size (Cohen’s *d* = +7.540). Nutritional risk (MNA-SF) scores remained stable (*p* = 0.102). (2) Psychosocial factors: Self-efficacy for chronic disease management increased progressively (*p* < 0.001; Cohen’s *d* = +1.805), with significant improvements in both gender subgroups. (3) Self-management behaviours: Scores for exercise-related and cognitive symptom management subscales increased significantly (both *p* < 0.001; Cohen’s *d* = +2.853 and +1.505, respectively). The communication subscale showed no significant change (*p* = 0.449). Self-reported exercise adherence was substantially higher during and after the intervention compared to baseline (*p* < 0.001; Cohen’s *d* = +2.551). (4) Digital engagement: Views of sarcopenia education videos on TikTok peaked after the initial education session and gradually declined, while views of exercise demonstration videos increased during the intervention phases and were sustained at follow-up (both overall *p* < 0.001). (5) Visual trends: The progression of these outcomes is illustrated in Supplementary Fig. 3. The plots show a sharp, sustained increase in knowledge, a gradual rise in self-efficacy, stability in nutritional risk, phase-aligned increases in exercise-related behaviours and adherence, and content-specific patterns in TikTok engagement.

**Table 5:** Changes of knowledge, psychosocial factors, and behavioural indicators from baseline to week 13

Variables	Assessment timepoint	M ± SD (95%CIs)		
		Total (*n* = 32)	Male (*n* = 7)	Female (*n* = 25)
Sarcopenia prevention quizzes	T_0_	4.63 ± 2.17 (3.84, 5.41)	4.14 ± 2.04 (2.26, 6.03)	4.76 ± 2.22 (3.84, 5.68)
	T_1_	19.22 ± 1.64 (18.63, 19.81)	19.00 ± 1.29 (17.81, 20.19)	19.28 ± 1.74 (18.56, 20.00)
	T_4_	19.81 ± 1.18 (19.39, 20.24)	19.43 ± 1.51 (18.03, 20.83)	19.92 ± 1.08 (19.48, 20.36)
	T_7_	20.50 ± 0.72 (20.24, 20.76)	20.43 ± 0.79 (19.70, 21.16)	20.52 ± 0.71 (20.23, 20.81)
	T_10_	20.59 ± 0.61 (20.37, 20.82)	20.29 ± 0.76 (19.59, 20.98)	20.68 ± 0.56 (20.45, 20.91)
	T_13_	20.81 ± 0.40 (20.67, 20.96)	20.71 ± 0.49 (20.26, 21.17)	20.84 ± 0.37 (20.69, 20.99)
Change (T_13_ – T_0_)				
MD ± SD (95%CI)		16.19 ± 2.15 (15.41, 16.96)	16.57 ± 1.72 (14.98, 18.16)	16.08 ± 2.27 (15.14, 17.02)
Cohen’s *d* (95%CI)		+7.540 (+5.638, +9.435)	+9.644 (+4.319, +15.012)	+7.079 (+5.047, +9.102)
*t*		42.654	25.517	35.394
*p*		<0.001	<0.001	<0.001
Mini-Nutritional Assessment Short Form	T_0_	13.38 ± 0.91 (13.05, 13.70)	13.71 ± 0.49 (13.26, 14.17)	13.28 ± 0.98 (12.88, 13.68)
	T_4_	13.25 ± 0.92 (12.92, 13.58)	13.71 ± 0.49 (13.26, 14.17)	13.12 ± 0.97 (12.72, 13.52)
	T_7_	13.34 ± 0.94 (13.01, 13.68)	13.71 ± 0.49 (13.26, 14.17)	13.24 ± 1.01 (12.82, 13.66)
	T_10_	13.50 ± 0.80 (13.21, 13.79)	13.86 ± 0.38 (13.51, 14.21)	13.40 ± 0.87 (13.04, 13.76)
	T_13_	13.50 ± 0.84 (13.20, 13.80)	13.86 ± 0.38 (13.51, 14.21)	13.40 ± 0.91 (13.02, 13.78)
Change (T_13_ – T_0_)				
MD ± SD (95%CI)		0.13 ± 0.42 (−0.03, 0.28)	0.14 ± 0.38 (−0.21, 0.49)	0.12 ± 0.44 (−0.06, 0.30)
Cohen’s *d* (95%CI)		+0.297 (−0.060, +0.649)	+0.378 (−0.406, +1.134)	+0.273 (−0.129, +0.670)
*t/Z*		−1.633^a^	−1.000^a^	−1.342^a^
*p*		0.102	0.317	0.180
Self-Efficacy for Managing Chronic Disease 6-item Scale	T_0_	29.56 ± 4.35 (28.00, 31.13)	28.57 ± 5.44 (23.54, 33.60)	29.84 ± 4.08 (28.16, 31.52)
	T_4_	30.00 ± 4.78 (28.28, 31.72)	28.29 ± 6.52 (22.25, 34.32)	30.48 ± 4.21 (28.74, 32.22)
	T_7_	31.75 ± 4.64 (30.08, 33.42)	29.71 ± 5.96 (24.20, 35.23)	32.32 ± 4.16 (30.60, 34.04)
	T_10_	33.09 ± 4.54 (31.46, 34.73)	31.14 ± 5.37 (26.18, 36.11)	33.64 ± 4.24 (31.89, 35.39)
	T_13_	34.34 ± 4.39 (32.76, 35.93)	32.29 ± 4.79 (27.86, 36.71)	34.92 ± 4.19 (33.19, 36.65)
Change (T_13_ – T_0_)				
MD ± SD (95%CI)		4.78 ± 2.65 (3.83, 5.74)	3.71 ± 3.20 (0.76, 6.67)	5.08 ± 2.47 (4.06, 6.10)
Cohen’s *d* (95%CI)		+1.805 (+1.234, +2.365)	+1.161 (+0.155, +2.115)	+2.061 (+1.354, +2.753)
*t*		10.212	3.071	10.304
*p*		<0.001	0.022	<0.001
Self-management Behaviour for Chronic Disease Scale (exercise)	T_0_	1.44 ± 0.84 (1.13, 1.74)	0.57 ± 0.53 (0.08, 1.07)	1.68 ± 0.75 (1.37, 1.99)
	T_4_	7.97 ± 2.25 (7.16, 8.78)	6.86 ± 0.90 (6.03, 7.69)	8.28 ± 2.42 (7.28, 9.28)
	T_7_	7.38 ± 1.70 (6.76, 7.99)	6.86 ± 1.21 (5.73, 7.98)	7.52 ± 1.81 (6.77, 8.27)
	T_10_	7.25 ± 1.93 (6.55, 7.95)	6.29 ± 0.49 (5.83, 6.74)	7.52 ± 2.10 (6.65, 8.39)
	T_13_	7.00 ± 1.80 (6.35, 7.65)	6.29 ± 0.49 (5.83, 6.74)	7.20 ± 1.98 (6.38, 8.02)
Change (T_13_ – T_0_)				
MD ± SD (95%CI)		5.56 ± 1.95 (4.86, 6.27)	5.71 ± 0.76 (5.02, 6.41)	5.52 ± 2.18 (4.62, 6.42)
Cohen’s *d* (95%CI)		+2.853 (+2.061, +3.635)	+7.559 (+3.355, +11.788)	+2.530 (+1.711, +3.336)
*t/Z*		−4.981^a^	20.000	−4.414^a^
*p*		<0.001	<0.001	<0.001
Self-management Behaviour for Chronic Disease Scale (cognitive)	T_0_	7.56 ± 2.68 (6.60, 8.53)	4.86 ± 1.07 (3.87, 5.85)	8.32 ± 2.50 (7.29, 9.35)
	T_4_	8.81 ± 3.13 (7.69, 9.94)	5.86 ± 1.57 (4.40, 7.31)	9.64 ± 2.96 (8.42, 10.86)
	T_7_	9.22 ± 3.44 (7.98, 10.46)	6.43 ± 1.72 (4.84, 8.02)	10.00 ± 3.42 (8.59, 11.41)
	T_10_	9.66 ± 3.45 (8.41, 10.90)	6.71 ± 1.11 (5.69, 7.74)	10.48 ± 3.44 (9.06, 11.90)
	T_13_	9.91 ± 3.19 (8.76, 11.06)	7.29 ± 1.60 (5.80, 8.77)	10.64 ± 3.15 (9.34, 11.94)
Change (T_13_ – T_0_)				
MD ± SD (95%CI)		2.34 ± 1.56 (1.78, 2.91)	2.43 ± 1.13 (1.38, 3.48)	2.32 ± 1.68 (1.63, 3.01)
Cohen’s *d* (95%CI)		+1.505 (+0.990, +2.008)	+2.142 (+0.728, +3.515)	+1.384 (+0.824, +1.929)
*t*		8.511	5.667	6.920
*p*		<0.001	0.001	<0.001
Self-management Behaviour for Chronic Disease Scale (communication)	T_0_	1.56 ± 1.13 (1.15, 1.97)	1.00 ± 1.15 (0.07, 2.07)	1.72 ± 1.10 (1.27, 2.17)
	T_4_	1.63 ± 1.04 (1.25, 2.00)	1.00 ± 1.15 (0.31, 1.98)	1.76 ± 1.05 (1.33, 2.19)
	T_7_	1.75 ± 0.95 (1.41, 2.09)	1.14 ± 0.38 (0.79, 1.49)	1.92 ± 1.00 (1.51, 2.33)
	T_10_	1.66 ± 0.79 (1.37, 1.94)	1.29 ± 0.76 (0.59, 1.98)	1.76 ± 0.78 (1.44, 2.08)
	T_13_	1.69 ± 0.90 (1.36, 2.01)	1.29 ± 0.49 (0.83, 1.74)	1.80 ± 0.96 (1.40, 2.20)
Change (T_13_ – T_0_)				
MD ± SD (95%CI)		0.13 ± 0.91 (−0.20, 0.45)	0.29 ± 0.76 (−0.41, 0.98)	0.08 ± 0.95 (−0.31, 0.47)
Cohen’s *d* (95%CI)		+0.138 (−0.221, +0.485)	+0.378 (−0.406, +1.134)	+0.084 (−0.310, +0.476)
*t/Z*		−0.758^a^	1.000	−0.393^a^
*p*		0.449	0.356	0.694
Exercise Adherence Rating Scale	T_0_	3.72 ± 2.14 (2.95, 4.49)	3.43 ± 2.23 (1.37, 5.49)	3.80 ± 2.16 (2.91, 4.69)
	T_4_	19.22 ± 3.61 (17.92, 20.52)	17.71 ± 3.30 (14.66, 20.77)	19.64 ± 3.64 (18.14, 21.14)
	T_7_	18.72 ± 3.78 (17.36, 20.08)	17.14 ± 2.61 (14.73, 19.56)	19.16 ± 3.98 (17.52, 20.80)
	T_10_	18.03 ± 4.40 (16.45, 19.62)	16.43 ± 4.79 (12.00, 20.86)	18.48 ± 4.27 (16.72, 20.24)
	T_13_	17.19 ± 4.56 (15.54, 18.83)	15.29 ± 4.03 (11.56, 19.01)	17.72 ± 4.63 (15.81, 19.63)
Change (T_13_ – T_0_)				
MD ± SD (95%CI)		13.47 ± 5.28 (11.57, 15.37)	11.86 ± 3.85 (8.30, 15.42)	13.92 ± 5.60 (11.61, 16.23)
Cohen’s *d* (95%CI)		+2.551 (+1.825, +3.267)	+3.081 (+1.219, +4.918)	+2.478 (+1.679, +3.282)
*t*		14.432	8.152	12.435
*p*		<0.001	<0.001	<0.001
Number of exposures to sarcopenia information videos on TikTok	T_0_	0.00 ± 0.00 (0.00, 0.00)	0.00 ± 0.00 (0.00, 0.00)	0.00 ± 0.00 (0.00, 0.00)
	T_1_	3.41 ± 0.62 (3.18, 3.63)	3.43 ± 0.79 (2.70, 4.16)	3.40 ± 0.58 (3.16, 3.64)
	T_4_	2.50 ± 0.72 (2.24, 2.76)	2.43 ± 0.54 (1.93, 2.92)	2.52 ± 0.77 (2.20, 2.84)
	T_7_	2.44 ± 0.72 (2.18, 2.70)	2.57 ± 0.79 (1.84, 3.30)	2.40 ± 0.71 (2.11, 2.69)
	T_10_	1.72 ± 0.77 (1.44, 2.00)	1.71 ± 0.49 (1.26, 2.17)	1.72 ± 0.84 (1.37, 2.07)
	T_13_	1.00 ± 0.80 (0.71, 1.29)	0.71 ± 0.76 (0.02, 1.41)	1.08 ± 0.81 (0.74, 1.42)
Change (T_13_ – T_0_)				
MD ± SD (95%CI)		1.00 ± 0.80 (0.71, 1.29)	0.71 ± 0.76 (0.02, 1.41)	1.08 ± 0.81 (0.74, 1.42)
Cohen’s *d* (95%CI)		+1.245 (+0.775, +1.703)	+0.945 (+0.014, +1.826)	+1.329 (+0.780, +1.864)
*t/Z*		−4.235^a^	2.500	−3.834^a^
*p*		<0.001	0.047	<0.001
Number of exposures to exercise information videos on TikTok	T_0_	0.22 ± 0.49 (0.04, 0.40)	0.00 ± 0.00 (0.00, 0.00)	0.28 ± 0.54 (0.06, 0.50)
	T_1_	0.25 ± 0.51 (0.07, 0.43)	0.00 ± 0.00 (0.00, 0.00)	0.32 ± 0.56 (0.09, 0.55)
	T_4_	1.63 ± 0.75 (1.35, 1.90)	1.14 ± 0.38 (0.79, 1.49)	1.76 ± 0.78 (1.44, 2.08)
	T_7_	2.16 ± 0.85 (1.85, 2.46)	1.71 ± 0.49 (1.26, 2.17)	2.28 ± 0.89 (1.91, 2.65)
	T_10_	1.94 ± 0.76 (1.66, 2.21)	1.57 ± 0.79 (0.84, 2.30)	2.04 ± 0.74 (1.74, 2.34)
	T_13_	1.94 ± 0.84 (1.63, 2.24)	1.57 ± 0.79 (0.84, 2.30)	2.04 ± 0.84 (1.69, 2.39)
Change (T_13_ – T_0_)				
MD ± SD (95%CI)		1.72 ± 1.02 (1.35, 2.09)	1.57 ± 0.79 (0.84, 2.30)	1.76 ± 1.09 (1.31, 2.21)
Cohen’s *d* (95%CI)		+1.679 (+1.132, +2.215)	+1.997 (+0.649, +3.303)	+1.613 (+1.006, +2.206)
*t/Z*		−4.768^a^	−2.414^a^	−4.155^a^
*p*		<0.001	0.016	<0.001

‘a’ means that the difference between T13 and T0 did not meet the normality assumption (Shapiro-Wilk test *p* < 0.05), thus the Wilcoxon signed-rank test was used. *T*_0_ Baseline, *T*_1_ Week 1 (health education), *T*_4_ Week 4 (exercise), *T*_7_ Week 7 (exercise), *T*_10_ Week 10 (follow-up), *T*_13_ Week 13 (follow-up). *MD* mean difference from baseline to week 13. The symbol ‘+’ denotes a positive direction of intervention effect, whereas the symbol ‘-’ indicates a negative direction of intervention effect. All p-values are unadjusted for multiple comparisons and should be interpreted as exploratory.

#### The monitoring of behaviour changes

**Fig. 3:**
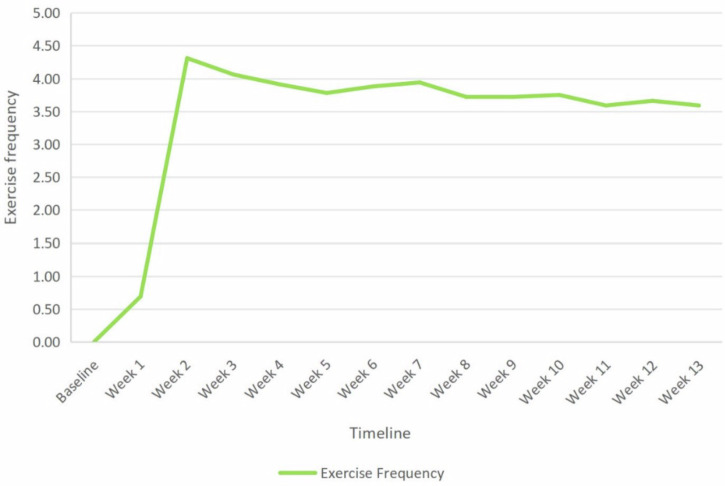
Mean weekly exercise frequency during the study period. Mean weekly exercise frequency was recorded based on weekly phone interviews conducted by research staff from baseline to week 13.

**Fig. 4:**
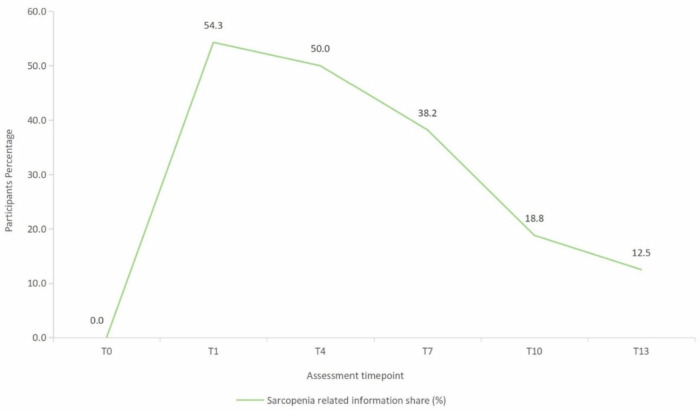
Participant engagement in sharing sarcopenia-related information. The percentage of participants who shared sarcopenia-related content with others during the intervention and follow-up period is shown.

Behaviour change was monitored across five domains: exercise adherence (self-reported ratings and diary records), digital engagement with intervention content on TikTok, information-sharing behaviours, and post-study exercise intentions. Quantitative adherence and digital exposure data are detailed in Table 5; additional behavioural findings are summarized below. (1) Exercise engagement patterns: Self-reported exercise adherence increased markedly during the intervention (see Table 5). Exercise diary data revealed that weekly exercise frequency rose from 0 at baseline to 4.31 ± 1.40 sessions by Week 2, then gradually declined to 3.59 ± 1.19 sessions by Week 13 (Fig. 3), suggesting a tapering trend in the unsupervised follow-up phase. (2) Information-sharing behaviour: The proportion of participants sharing sarcopenia-related information peaked at 54.3% during the initial health education week (T1), then steadily decreased to 12.5% by the final follow-up (T13; Fig. 4). (3) Post-Study Exercise intentions: All participants (100%) expressed intention to continue exercising after the study. Of these, 75% (n = 24) planned to exercise at least three times per week and increase as much as possible, while 25% (*n* = 8) reported they would exercise as their schedule allowed, without a fixed weekly routine.

### Semi-structured interview

**Fig. 5:**
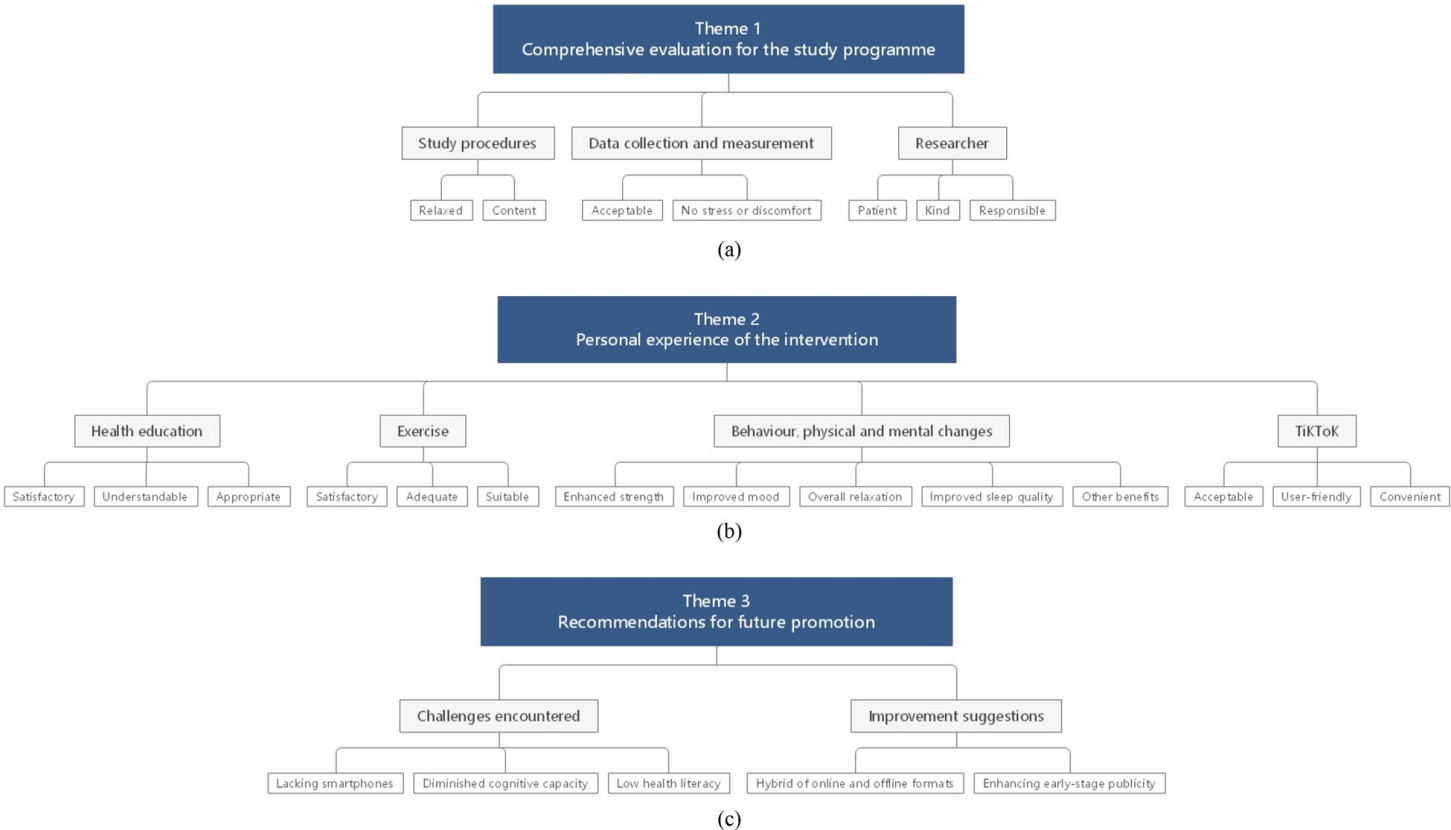
Themes identified from semi-structured interviews. This figure summarises key themes and subthemes derived from qualitative analysis of participant interviews exploring experiences, perceived benefits, and acceptability of the SHEEP intervention. **a** presents Theme 1 “Comprehensive evaluation for the study programme”; **b** presents Theme 2 “Personal experience of the intervention”; **c** presents Theme 3 “Recommendations for future promotion”.

Semi-structured interviews were conducted upon study completion among 26 participants (mean duration: 23.27 ± 7.09 minutes). The interviews were categorised into three primary themes and eight sub-themes: the comprehensive evaluation for this research (study procedures, data collection and measurement, researcher), the participant’s personal experience of the intervention (health education; exercise; behaviour, physical and mental changes), and recommendations for future promotion (challenges encountered, improvement suggestions) as shown in Fig. 5. Some examples of interview responses are summarized according to different themes, as detailed in Supplementary Note 2.

#### Theme 1: participants’ comprehensive evaluation of this research

##### Study procedures

All 26 participants expressed satisfaction with their involvement in the study and reported feeling at ease and content throughout its duration.

##### Data collection and measurement

The 26 participants concurred that the data collecting, and measurement processes were acceptable, with no reported stress or discomfort.

##### Researcher

Fourteen (53.85%) participants actively mentioned and expressed favourable opinions of the PI, such as being patient, kind and responsible.

#### Theme 2: participants’ personal experience of the intervention

##### Health education

Using the Likert-5 score, 25 (96.15%) participants rated the health education strategy as very satisfactory, with one (3.85%) rating it as satisfactory. The evaluation of health education strategy was categorised in four aspects: health education content, duration, frequency, and delivery tool.

① Health education content: All participants reportedly found the health education information easy to understand and said that they benefited from the knowledge of sarcopenia prevention to varying degrees.

② Health education duration: 23 (88.46%) participants believed 4-6 minutes per video was appropriate, but three participants thought it could be extended a bit, as they wished to gain more regarding sarcopenia prevention.

③ Health education frequency: participants exhibited variability in the frequency of video viewership during the initial week. Twelve (46.15%) participants reported viewing 1-2 videos at a time, five (19.23%) indicated they preferred to watch all videos in one go, while the remaining nine (34.62%) participants stated that their viewing quantity was contingent upon their mood, but they would complete all videos within the first week.

④ Delivery tool for health education: all participants deemed the acquisition of health education knowledge via TikTok to be acceptable and user-friendly.

##### Exercise intervention

Using the Likert-5 score, all participants reported their overall assessment of the exercise strategy as being very satisfied. The evaluation can be distilled into five aspects: exercise content, duration, frequency, promotional film, and delivery tool.

① Exercise content: all participants deemed the exercise content satisfactory and the movement design adequate. Using water bottles for resistance exercise was considered novel by the participants. Furthermore, all participants reported no adverse reactions or discomfort during the exercise.

② Exercise duration: 22 (84.62%) participants stated that an exercise duration of 25 to 30 minutes was suitable, while the remaining 4 (15.38%) individuals deemed it acceptable to moderately extend the duration.

③ Exercise frequency: all participants deemed a frequency of doing exercise at least three times weekly to be entirely attainable.

④ Promotional films about exercise: all participants agreed that the promotional films about resistance exercise for sarcopenia prevention contributed to increasing their awareness of sarcopenia prevention and improving their motivation to exercise.

⑤ Delivery tool of exercise: all participants deemed the use of TikTok for exercise acceptable, citing advantages such as independence of time, location, and climate.

##### Behaviour, physical and mental changes

All participants reported experiencing varying degrees of benefit to their subjective feelings, encompassing behaviour, physical, and psychological aspects. Following the exercise intervention, 25 (96.15%) participants reported enhanced strength in their hands and feet, 18 (69.23%) noted an improved mood, 17 (65.38%) experienced greater overall relaxation of the whole body, 16 (61.54%) indicated improved sleep quality, 12 (46.15%) reportedly altered their diet, including increased protein consumption, eight (30.77%) reported an enhanced appetite, five (19.23%) indicated an enhancement in gastrointestinal function, including alleviation of constipation, and four (15.38%) said they had increased energy levels when doing chores in their daily life. In addition, all participants expressed their willingness to learn more about sarcopenia after finishing this project, and to persist in exercising to prevent sarcopenia, and to disseminate information regarding sarcopenia to their family and friends.

#### Theme 3: recommendations for future promotion

Ten participants identified obstacles and dilemmas regarding future promotion of this study. For instance, there are barriers in promoting it to older adults without smartphones, or who have diminished cognitive capacity or low health literacy. Three participants proposed potential solutions, such as creating a hybrid of online and offline formats and enhancing early-stage publicity.

## Discussion

This single-arm feasibility study demonstrates that SHEEP and its associated trial procedures were feasible and acceptable for community-dwelling older adults with possible sarcopenia. Exploratory analyses of secondary outcomes showed promising trends toward improvement in handgrip strength, walking speed, sit-to-stand performance, sarcopenia prevention knowledge, self-efficacy, self-management behaviours, and exercise adherence. The preliminary estimates of effect size and variability derived from these data provide essential parameters for informing the design and sample size calculation of a future definitive randomised controlled trial.

The single-arm, pre-post design was selected primarily due to constraints in time and research personnel, which aligned with the core objectives of this feasibility study. A limited recruitment window (2–3 weeks) and management by a single investigator made targeting a sample of 35 participants pragmatic for a first test of procedures; recruiting a larger cohort sufficient for a feasibility RCT (e.g., 60–70 participants) was not viable under these resource conditions. This pragmatic design efficiently allowed us to address the key uncertainties preceding a definitive trial: namely, the feasibility of recruitment, intervention delivery, and outcome assessment procedures within the intended community setting. For recruitment, the two-step screening method (SARC-CalF questionnaire followed by handgrip strength measurement) proved feasible and consistent with international guidelines (AWGS2, EWGSOP2) that prioritize low muscle strength for identifying possible sarcopenia in community settings^2,3^. While more comprehensive diagnostic tools exist (e.g., bioelectrical impedance analysis), our pragmatic two-step method effectively identified older adults likely to benefit from prevention, balancing adequate accuracy with practical implementation in a resource-limited community setting.

Regarding recruitment, while the target sample size was achieved, the sample was not fully representative. A marked age imbalance was observed, with 80.0% of participants aged 65–69 years compared to 20.0% aged 60–64 years. This pattern, which was also present in our prior pilot work (70.0% vs 30.0%)^32^, suggests a potentially higher prevalence or detection rate of possible sarcopenia in the older cohort, although this warrants confirmation in larger, age-stratified epidemiological studies. Furthermore, a pronounced gender imbalance existed (80% female, 20% male). This under-representation of men, a common challenge in community-based sarcopenia research^20,43–45^, limits the generalizability of our findings to older males. These imbalances likely arose from a combination of methodological and contextual factors. Our recruitment relied on three primary channels: electronic leaflets, paper leaflets, and word-of-mouth. While electronic dissemination proved most effective, this approach may have inadvertently reached a narrower, more digitally engaged subset of the community. Furthermore, recruitment outcomes varied substantially between the two communities (Sanchaji: *n* = 33; Guanshaling: *n* = 2), potentially due to the presence of a dedicated senior activity centre in the former, which served as a natural hub for information sharing and trust-building.

To ensure a more representative sample in a future definitive trial, a multi-faceted recruitment strategy is required, one that directly addresses the specific imbalances observed in this feasibility study. Proactive, targeted efforts must complement broad-based outreach. First, to directly address the pronounced under-representation of men (only 20% of our sample), recruitment should be strategically embedded in settings frequented by older men who may be at risk due to sedentary lifestyles, such as card, mahjong clubs, or teahouses, with messaging that emphasizes preserving strength for autonomy and family roles rather than generic ‘exercise.’ Second, to mitigate the substantial variation in recruitment yield observed between our two study communities (Sanchaji: *n* = 33; Guanshaling: *n* = 2), collaboration with community health service centres should be deepened, moving beyond passive leaflet distribution to co-hosting educational talks or health fairs, thereby leveraging their institutional trust. Third, building on the observation that the presence of a dedicated senior activity centre appeared to facilitate recruitment in one community, site selection must consider community infrastructure; partnering with communities that have active senior centres may improve efficiency, while additional resources and tailored approaches (e.g., door-to-door outreach, community leader involvement) should be allocated for settings lacking such facilities. Ultimately, a hybrid recruitment model, blending digital convenience, trusted community partnerships, and targeted in-person engagement, is recommended to overcome the limitations observed in this feasibility study.

The research procedures demonstrated high acceptability. All interviewed participants expressed satisfaction, and the mean assessment duration decreased slightly from baseline to final follow-up (36.21 ± 2.59 to 35.37 ± 2.50 minutes), likely reflecting increased familiarity with the protocol. Participant adherence was excellent: 100% completed health education video viewing, 97.1% initiated the exercise programme, and no exercise-related adverse events were reported, contributing to a high retention rate (91.4%). This high engagement can be attributed to two key factors. First, the intervention itself was highly rated for its content, format, and flexibility, qualities informed by its co-design development and the inherent advantages of the social media delivery platform^31,32^. Second, participants explicitly valued the supportive and communicative approach of the research team, contrasting it with often time-constrained clinical interactions^46–49^. This underscores that for a digital preventive intervention, the combination of a well-designed program and proactive human support may be a critical driver of feasibility and retention in future trials.

Operational feasibility required adaptive timeline management, yielding key logistical insights. Recruitment took three weeks rather than the planned two, even with prior community experience, indicating that future trials in new community settings should allocate a minimum of 3–4 weeks for recruitment to secure a representative sample. This initial delay also created a cascade effect, severely compressing the time window for qualitative interviews due to a fixed ethics deadline, which prevented six participants from being interviewed. This experience highlights that feasibility encompasses not only participant adherence but also robust operational planning with built-in buffers for unpredictable phases^50^. Furthermore, recruitment channels must align with the target population’s habits (e.g., prioritising WeChat over email). These practical lessons on timeline flexibility, resource allocation, and community-adapted methods are crucial for the executable design of a subsequent RCT.

Exploratory analyses of the key sarcopenia-related domains indicated directional trends. For functional capacity outcomes, improvements were observed in handgrip strength (both hands), 4-meter walking speed, and sit-to-stand time from baseline to week 13 in the total sample, with large within-group effect sizes. These trends were generally consistent across gender subgroups. The pattern of improvement in handgrip strength aligns with outcomes reported in some trials of digital interventions for sarcopenia^25,51–53^, whereas changes in walking speed^51,52^ and sit-to-stand ability^54,55^ are less consistently reported. Moreover, the body composition data presented a mixed profile. While total skeletal muscle mass remained stable, regional increase trends were noted in trunk and upper-extremity skeletal muscle mass and upper arm dimensions, which may reflect the focus of the resistance training component. This discrepancy—between stable overall mass and regional gains—exemplifies the inconsistent findings prevalent in digital interventions for sarcopenia regarding changes in skeletal muscle mass^25,51,53,54,56,57^. This inconsistency is highlighted in our recent meta-analysis, which suggests that while such interventions may not significantly improve the appendicular skeletal muscle index, they could positively affect total skeletal muscle mass^30^. However, the overall quality of the existing evidence is low, underscoring the need for more rigorously designed trials to clarify these effects^30^. Concurrently, increases in body weight, BMI, and total body fat were observed. These concurrent changes, which find both parallels^53–55^ and contradictions^51,56^ in other digital sarcopenia interventions, underscore the inherent complexity of modulating body composition. They suggest that integrating a more structured nutritional component may be crucial in future intervention designs. All body composition findings should be interpreted as generating hypotheses regarding potential intervention effects rather than as evidence of efficacy.

Changes in participant-reported outcomes and monitored behaviours were observed. An increase in sarcopenia prevention knowledge scores was recorded following the initial education session and was sustained, a relevant finding in the context of widely reported low awareness of this condition^58–61^. This was accompanied by increases in scores for self-efficacy for managing chronic disease and for specific self-management behaviours (e.g., exercise, cognitive symptom management), alongside greater self-reported exercise adherence during the active intervention phases. The co-occurrence of knowledge gain with these psychosocial and behavioural trends is consistent with the pathways proposed by health behaviour theories^62–64^. Engagement with the digital intervention components, as reflected in usage metrics and behavioural monitoring data (e.g., exercise diaries, information-sharing), further indicates that the delivery model was feasible for engaging participants. However, the interpretation of these outcomes is circumscribed by methodological considerations. The generic scales employed, including the Self-Efficacy for Managing Chronic Disease 6-item Scale and the Self-Management Behaviour for Chronic Disease Scale^65–67^, are not specific to sarcopenia and may not optimally capture relevant self-efficacy constructs or behaviours (e.g., resistance exercise, protein intake management). Furthermore, participants reported perceived improvements in physical and mental well-being in qualitative interviews, such as increased strength, better mood, improved sleep, and higher energy levels. These subjective accounts align with the multi-system benefits often associated with exercise interventions^68^.

The apparent discrepancy between the declining exercise frequency recorded in diaries (from 4.31 to 3.59 sessions/week) and high satisfaction reported qualitatively can be reconciled through a multi-faceted interpretation. First, satisfaction likely reflects the intervention’s acceptability and perceived utility (e.g., home-based flexibility), which is distinct from strict adherence to a prescribed dose. Second, the maintained frequency (3.59 sessions/week) still met the program’s minimum target, suggesting participants may have adopted a sustainable, personalized routine they valued positively. This aligns with a cultural context where older adults often integrate physical activity into daily life rather than segregating it as a formal ‘exercise’ regimen. Third, satisfaction may be driven by benefits not fully captured by session counts, such as enhanced self-efficacy, enjoyment, or perceived improvements in well-being and strength noted in interviews. Finally, the gradual decline itself underscores a common challenge in behavioural maintenance within unsupervised interventions^69,70^, potentially due to waning novelty or competing priorities.

A similar pattern of declining engagement was observed in information-sharing behaviours, which peaked during the initial health education week (54.3%) and gradually decreased to 12.5% by the final follow-up. This trend is consistent with previous research on digital health interventions, which has shown that user engagement, particularly in peer-to-peer sharing and social features, often declines over time after an initial peak^71,72^. This likely reflects the natural trajectory of dissemination, where initial enthusiasm and novelty drive active sharing, followed by a stabilisation or habituation phase^73^. However, it also highlights a potential limitation in sustaining social engagement around sarcopenia prevention once the structured intervention ends.

Given that social support and peer influence are well-established facilitators of long-term behaviour change^74,75^, strategies to sustain exercise frequency and information-sharing warrant consideration in future interventions. Approaches such as creating online community groups, organising periodic peer-led discussions, or sending prompts with new educational content have shown promise in maintaining social engagement in other health promotion contexts^76–78^. These strategies may help sustain both individual motivation and collective engagement^79,80^. Moving forward, longer intervention periods integrated with behavioural maintenance strategies, such as booster sessions^81^, social support mechanisms^82^, or adaptive goal-setting^83^, are recommended to help solidify exercise habits and fully realise physiological benefits, while continuing to respect participants’ preference for sustainable integration over rigid adherence. These strategies may also serve to sustain social engagement and information-sharing, thereby reinforcing the social norms that support healthy behaviours in community settings^84^.

This feasibility study has several limitations. First, its single-arm, exploratory design with multiple comparisons precludes causal efficacy inferences and increases Type I error risk, necessitating a future RCT. Second, generalizability is limited by the sample’s origin from only two communities, pronounced gender imbalance (80% female), and small size, which also affects the precision of effect estimates for future trial planning. Third, while the retention rate was high (91.4%), the withdrawal of three participants and the use of complete-case analysis for secondary outcomes may influence efficiency and bias; future trials should implement more rigorous strategies to collect outcome data from withdrawers. Fourth, exercise adherence was primarily assessed using self-reported diaries and scales, which are susceptible to recall and social desirability biases, a recognised limitation in behavioural intervention research. To address this, future definitive trials should incorporate objective adherence measures, such as accelerometer-based wearables or digital activity trackers, to validate self-reported data and provide a more accurate assessment of exercise dose and compliance. Fifth, the intervention excluded older adults reliant on mobility aids (e.g., wheelchairs) and those without internet access, limiting inclusivity. To address the digital divide and enhance the reach of future interventions, a hybrid (online-offline) delivery model is recommended. Core strategies should focus on leveraging community infrastructure, such as utilizing multimedia systems in community centres for group sessions, and adopting a tiered, flexible participation framework. This could combine facilitated, in-person core sessions with supplementary at-home practice using diverse materials (e.g., online videos, illustrated handouts). Such an approach aims to preserve the intervention’s key components while pragmatically overcoming barriers related to access, digital literacy, and participant preference.

In summary, this study provided preliminary evidence that delivering a social media-based multicomponent intervention (SHEEP) to community-dwelling older adults with possible sarcopenia was feasible and safe, with respect to recruitment, procedures, assessment, and intervention delivery. The research processes were also found to be acceptable to both participants and researchers. As the first study to explore the operability of such an intervention in a real-world setting, it offers preliminary insights into implementing TikTok-based health education and exercise within communities. Exploratory analyses also indicated directional trends in several secondary outcomes (e.g., physical function, knowledge, self-efficacy). These trends should be interpreted as hypothesis-generating; the accompanying effect size estimates are presented solely to inform the design of a future randomised controlled trial and must be interpreted with caution due to the uncontrolled design and small sample size. The findings collectively highlight key considerations for future research, including recruitment strategies, safety monitoring, and intervention sustainability.

Building on these insights, the feasibility data also provide concrete parameters to inform the design of a future definitive RCT. Based on the exploratory analysis of secondary outcomes, handgrip strength emerges as the most suitable primary outcome, given its direct relevance to the definition of possible sarcopenia^2,3^. The sample size was calculated for a two-arm parallel-group RCT comparing changes in handgrip strength between groups. To avoid overestimation of treatment effects commonly observed in pilot studies, a conservative minimal clinically important difference (MCID) of 2.5 kg was specified for the between-group difference. The standard deviation of the change score was assumed to be 2.5 kg, slightly inflated from the observed 2.32 kg to account for uncertainty. Using a two-sample t-test with a two-sided significance level of 0.05 and 90% power, the required sample size was estimated to be 21 participants per group. Allowing for an anticipated attrition rate of 10%, the final target sample size is 24 participants per group (48 in total). This calculation is based on a comparison of change scores; in practice, an ANCOVA model adjusting for baseline values may improve statistical efficiency, suggesting that the present estimate is conservative.

## Methods

### Study design and reporting

The study employs a single-arm prospective pre-post design to assess the feasibility, acceptability, and preliminary impact of the SHEEP on preventing sarcopenia in community-dwelling young-old individuals with possible sarcopenia. Both quantitative and qualitative methods are employed to evaluate the research outcomes. This study is reported in accordance with the CONSORT extension to pilot and feasibility trials^85^ (excluding items that are specific to the randomisation nature of the study) and the Guidelines for Reporting Outcomes in Trial Reports (the CONSORT-Outcomes 2022 Extension)^86^. We also refer to the guidelines for reporting non-randomised pilot and feasibility studies proposed by Lancaster et al.^87^. This study was registered at the ISRCTN registry (ISRCTN17269170; 14 September 2023; 10.1186/ISRCTN17269170). The full study protocol has been published previously^88^.

### Research ethics

This study was conducted in accordance with the Declaration of Helsinki. Ethical approval was obtained from the University of Manchester Research Ethics Committee (Project ID: 2024-19302-34066). Appropriate permissions were also granted by the two collaborating community health centres and the Community Nursing Department of Xiang Ya Nursing School, Central South University, China. Written informed consent to participate in the study was obtained from all participants prior to data collection. No identifiable images or personal data are presented in this manuscript.

### Research setting

This feasibility study was conducted in Changsha City, Hunan Province, China. The recruitment and intervention procedures were carried out in two communities: Sanchaji Community and Guanshaling Community.

### Participants

This feasibility study targeted community-dwelling young-old adults (60–69 years) with possible sarcopenia. The screening protocol, comprising the SARC-CalF questionnaire followed by handgrip strength measurement, was selected in accordance with the AWGS2 guidelines for identifying possible sarcopenia in community settings^3^, as it efficiently identifies individuals with low muscle strength, the core criterion for possible sarcopenia, using feasible, low-cost tools suitable for community screening^89^. Detailed inclusion and exclusion criteria are presented in Table 6.

**Table 6:** Eligibility criteria of participants

Inclusion criteria	Exclusion criteria
1) 60 ~ 69 years old	1) Unable to communicate or independently complete learning and exercise using TikTok
2) Chinese residents living in the community	2) Serious or unstable medical illness, such as severe cardiovascular or respiratory conditions, mental disorder, dementia, etc.
3) Have a smartphone with internet connection at home, existing users of TikTok or willing to download and use TikTok	3) Currently undertaking regular exercise (e.g., Tai Chi or Square Dance) and exercise intensity is moderate (≥ 150 mins/week)
4) Individuals screened by five-item sarcopenia screening questionnaire: (strength, walking assistance, rising from a chair, stair-climbing, and falls) combined with calf circumference [SARC-CalF] ≥11	4) Contraindications for the use of Bioelectrical Impedance Analysis (BIA), such as people with implanted cardiac pacemakers, or other electronic devices or metal grafts, or with significant pitting oedema, or with limb dysfunction or body paralysis, or taking medications that affect body composition, such as diuretics or glucocorticoids.
5) Individuals with possible sarcopenia, as defined by low Grip Strength [M: < 28 kg, F: < 18 kg] in accordance with the 2019 Asian working group consensus on diagnosis criteria for sarcopenia	
6) Able to attend the community health centre (at their own expense if travel is required) on six occasions for the duration of the study	
7) Informed consent for screening and research	

### Sample size

The target sample size of 35 participants was determined based on methodological recommendations for pilot and feasibility studies, rather than a formal power calculation for hypothesis testing. While a minimum of 12 participants per group is often cited as sufficient for estimating key parameters such as standard deviation and attrition^90^, more recent guidance by Whitehead et al.^91^ proposes a stepped approach. They recommend that pilot trial sample sizes should be scaled according to the anticipated standardised effect size for the main trial. For expected small to medium effect sizes (e.g., δ between 0.1 and 0.7), the recommended pilot sample size ranges from approximately 10 to 25 participants per arm. Although these recommendations were originally developed for two-arm randomised trials, a total sample size of 35 is generally regarded as adequate for parameter estimation in a single-arm feasibility study and is broadly consistent with this guidance. It provides a robust basis for estimating the variability of key outcomes (e.g., handgrip strength, gait speed) to inform sample size calculations for a future definitive randomised controlled trial. This sample size also accounted for pragmatic considerations, including the single-centre recruitment context, available resources, and the need to assess the feasibility of recruitment, intervention delivery, and study procedures within a limited timeframe.

### Recruitment, consent, and withdrawal

The principal investigator (PI) visited the two community health centres to present the research to the managers and obtain their agreement. The PI explained the eligibility criteria for participation given in the information leaflets. The managers facilitated the distribution of printed informational leaflets to older people visiting community health centres and disseminated electronic recruitment leaflets to community WeChat groups. Older persons expressed their interest in this study by contacting the PI through the managers or using the contact information provided in the leaflet. In accordance with the inclusion and exclusion criteria, the PI conducted an initial eligibility screening via phone or email, and upon confirmation, carried out assessments in an office provided by the community health centre. Eligible participants received a comprehensive explanation of the research along with a participant information sheet and were afforded a minimum of 24 hours to decide upon participation. Informed consent was obtained prior to any study procedures. Participants provided either written consent or witnessed verbal consent (via telephone or in-person). For verbal consent, the PI read the full consent form aloud. The participant’s agreement to each key element (e.g., voluntary participation, confidentiality, right to withdraw) was confirmed verbally. This verbal consent was formally documented using a standardised consent record form, which included the participant’s name, the date, a statement of consent, and the signature of the researcher who obtained the consent. This form was stored with the written consent documents in accordance with data protection regulations. Participants were allowed to withdraw at any time without explanation.

### Intervention

Participants received one week’s health education, six weeks’ exercise training, and a six-week follow-up. (1) Health education: Participants viewed seven health education videos (4-6 minutes each) concerning sarcopenia prevention, which were posted on TikTok (TikTok ID: 73296723633) throughout the initial week. Participants faced no limitations on the number of views, permitting them to decide the number of times they viewed videos daily. (2) Physical exercise: Participants followed a structured, 30-minute exercise programme delivered via TikTok, comprising four components: warm-up (3 minutes), aerobic training (8 minutes), resistance training (16 minutes), and flexibility training (3 minutes). The prescribed exercise frequency was a minimum of three sessions per week. Exercise intensity was targeted at a moderate level, corresponding to a rating of 3–4 on the Borg Category-Ratio 10 (CR-10) Scale^92^. Resistance training was performed using adjustable plastic water bottles as weights (initial options: 500 ml or 1000 ml). A standardised progression protocol was applied: participants increased the load by adding 100–500 ml of water to each bottle every three weeks, based on their individual tolerance and perceived exertion. A detailed description of the exercise prescription, including specific movements for each component, is provided in Table 7, reported in accordance with the TIDieR guidelines^93^. (3) Follow-up: Participants were monitored for six weeks, during which the researcher assisted them in completing their activity diaries and documenting their feelings through brief telephone calls or text messages each week.

**Table 7:** Detailed exercise prescription of the SHEEP Programme

Exercise type	Duration	Exercise Contents
	30 min	
Warm-up training	3 min	1. Marching: 30 s
		2. Side taps: 60 s
		3. Shoulder circles: 60 s
		4. Trunk twist: 60 s
Aerobic training	8 min	1. Marches and bigger: 30 s
		2. Side steps: 60 s
		3. Stride with chest expansion: 60 s
		4. High leg lifts (two sets): 60 s in total
		5. 15 s in first set + 15 s marching (relax) + 15 s in second set + 15 s marching (relax)
		6. Stride with arms raised: 60 s
		7. Simple jumping jacks: 60 s
		8. Marches and bigger: 30 s
Resistance training	16 min	1. Weight squat: 6–12 times/set; 1–2set; relax (15-30 s)
		2. Upward push: 10–15 times/set; 1-2set; relax (15-30 s)
		3. Weight squat and upward push: 6–12 times/set; 1–2set; relax (15–30 s)
		4. Side shoulder raise: 10–15 times/set; 1–2set; relax (15–30 s)
		5. Arm forward and up: 10–15 times/set; 1–2set; relax (15–30 s)
		6. Relax: 2 min (e.g., drink water)
		(**Progressive with 500** **ml, 1000** **ml, 1500** **ml, 2000ml weight of mineral water bottles**).
		Note: Participants can start from 500 ml or 1000 ml, then add 500 ml of weight every three weeks. However, the increase in weight is not fixed, which can be adjusted according to the actual situation. When the body feels no burden after the whole exercise, for instance the body does not heat, sweat or muscles do not appear slightly sore after exercise, we suggest appropriately increasing the weight (100-500 ml).
Flexibility training	3 min	1. Back of upper arm stretch: 60 s in total; 2sets/each arm (15 s/set)
		2. Side bends: 60 s in total; 2sets/each side (15 s/set)
		3. Inner thighs stretch: 60 s in total; 2sets/each leg (15 s/set)
**Summary**		Contents: Four exercise types
		Frequency: ≥ 3days/week (Progressive)
		Duration: 30 minutes/day
		Intensity: Moderate
		Format: Video (Motion display with oral description)

### Outcomes

Measurements included socio-demographic information, primary outcomes, secondary outcomes, and exercise adherence. In brief, the primary outcomes involved evaluating five domains. (1) Recruitment capability and participant characteristics; (2) Intervention and study procedures’ acceptability and suitability; (3) Data collection procedures and outcome measurements; (4) Research duration and the ability of researcher to conduct the study and intervention; (5) Participant responsiveness to intervention. Consistent with established guidelines for pilot and feasibility trials^94^, we a priori defined quantitative progression criteria for three key process outcomes. These criteria were informed by methodological recommendations and benchmarks used in comparable studies of behavioural interventions in older adults^95,96^: (1) Recruitment rate: success was defined as $\ge$50% of approached eligible individuals providing consent to participate. (2) Intervention adherence rate: success was defined as $\ge$70% of participants completing at least 80% of the supervised exercise sessions. (3) Retention Rate: success was defined as $\ge$70% of participants completing all scheduled follow-up assessments. The evaluation of feasibility and the decision regarding progression to a future definitive randomized controlled trial were based on these pre-specified criteria. Progression was planned if all three criteria were met. If one or two criteria were not met, a detailed analysis of the underlying causes would be conducted to inform specific modifications to the trial protocol (e.g., refining recruitment strategies, enhancing participant support for adherence and retention, or adjusting intervention components). If none of the criteria were met, the trial would be deemed not feasible in its current form.

The secondary outcomes assessed two main categories: (1) Muscle-related outcomes: muscle strength (handgrip strength), muscle mass (skeletal muscle mass, skeletal mass index, upper-extremity skeletal muscle mass, lower-extremity skeletal muscle mass, and trunk skeletal muscle mass), and physical performance (walking speed and sit-to-stand function); (2) Other measurements: other body parameters (e.g., height, weight, calf circumference, abdominal circumference, body fat, body mass index, protein, and bone mineral), questionnaires (e.g., nutrition state, perceived knowledge, personal motivation, behavioural skills, and monitoring of behaviour change). All outcome measures were administered by the PI to ensure consistency and compliance with the ethical data protection protocol. To further enhance reliability, measures requiring repeated assessments, specifically handgrip strength and 4‑metre gait speed, were performed three times per session in accordance with established protocols^3,88^. For handgrip strength, the maximum value of the three trials was recorded; for gait speed, the mean time was calculated and used in the analysis.

Adherence was assessed using four methods: (1) Exercise adherence rating scale: a self-reported questionnaire administered at each measurement timepoint; (2) Exercise diaries: participants maintained weekly self-reported diaries of exercise frequency and duration; (3) Objective digital engagement: The backend data of TikTok provided objective metrics on the number of exposures (views) to both the health education and exercise instruction videos for each participant. (4) Behavioural metrics: self-reported sharing of information and future exercise intentions were collected via questionnaire. We explicitly acknowledge that the primary measures of exercise performance (frequency, duration, intensity) relied on self-report (diaries and scales), which are susceptible to recall and social desirability biases. The objective TikTok analytics served to corroborate engagement with the digital content, but did not directly measure physical activity execution. Table 8 delineates the diverse instruments used and the various time points at which the outcomes were evaluated during the research. Study protocol delineated the details of outcomes and diverse instruments used during the research^88^.

**Table 8:** Overview of instruments and time of outcome measures

Measures	Instruments	Assessment timepoint					
		T_0_	Intervention			Follow-up	
			T_1_	T_4_	T_7_	T_10_	T_13_
Social-demographic information	Demographics questionnaire	X					
**Primary outcome**							
Feasibility and acceptability	Research record sheet						
**Secondary outcome**							
Handgrip strength	Digital hand-held dynamometer	X		X	X	X	X
Anthropometric measurements (e.g. weight, height)	Stadiometer + Scale + Plastic tape	X		X	X	X	X
Body composition (e.g., muscle mass, body fat)	Bioelectrical impedance analyser device	X		X	X	X	X
Physical performance	4-Metre Walk Test	X		X	X	X	X
	Five Times Sit to Stand Test	X		X	X	X	X
Nutrition state	Mini-Nutritional Assessment Short Form	X		X	X	X	X
Perceived knowledge	21 true or false quizzes related to sarcopenia	X	X	X	X	X	X
Personal motivation	Self-efficacy for Managing Chronic Disease 6-item Scale	X		X	X	X	X
Behavioural skills	Self-management Behaviour for Chronic Disease Scale	X		X	X	X	X
Behaviour changes monitoring	Exercise diary (Every week)						
	Exercise Adherence Rating Scale	X		X	X	X	X
	Records of sharing sarcopenia-related information	X	X	X	X	X	X
	Willingness to formulate habits of regular exercise after study						X
	Records of exposure percentage of exercise and sarcopenia related videos	X	X	X	X	X	X

*T*_0_ Baseline, *T*_1_ Week 1 (health education), *T*_4_ Week 4 (exercise), T_7_ Week 7 (exercise), *T*_*10*_ Week 10 (follow-up), *T*_13_ Week 13 (follow-up).

### Safety monitoring and adverse events

To ensure participant safety during the unsupervised home-based exercise, a proactive monitoring procedure was implemented. The PI contacted each participant weekly via brief telephone calls or text messages. These contacts served to (1) remind participants to report their activity diaries, (2) enquire about any discomfort, adverse events (e.g., muscle soreness, pain, falls), or difficulties with the exercises, and (3) provide motivational support. Participants were instructed to report any concerns immediately.

### Statistical analysis

All quantitative analyses were conducted using IBM SPSS Statistics (Version 27.0). Primary feasibility outcomes (recruitment, retention, adherence, and acceptability) were summarised using descriptive statistics. Proportions were reported with 95% confidence intervals (CIs; Wilson score method), and continuous variables as mean ± standard deviation or median and interquartile range, as appropriate. These statistics were used to assess whether pre-defined feasibility benchmarks were met^94–96^. Analyses of secondary outcomes aimed to estimate parameters (e.g., means, variability) for future trial planning, not to test hypotheses. For continuous outcomes, within-participant change from baseline was quantified using: (1) the mean difference with 95% CI (paired methods); and (2) the within-subject effect size (Cohen’s d_z, calculated as M_diff/SD_diff)^97,98^ between baseline and final follow-up. The magnitude of d_z was interpreted as: 0.2 = small, 0.5 = medium, 0.8=large, 1.3 = very large^99^. Exploratory p-values from paired t-tests or Wilcoxon signed-rank tests are reported unadjusted for multiple comparisons to describe trends only^87,100^. Primary feasibility analyses followed the intention-to-treat principle^85^, including all consented participants (*n* = 35). Exploratory analyses of secondary outcomes used a complete-case approach, including participants with valid data at the relevant time points (*n* = 32).

Semi-structured interviews were conducted with study completers (*n* = 26) to explore their experiences and perceptions of the SHEEP programme. Following verbatim transcription of audio recordings, the qualitative data were analysed using reflexive thematic analysis, adhering to the six-phase approach by Braun and Clarke^101^. One researcher (Y.S.) performed the primary coding and preliminary theme development using NVivo 12 software. To enhance analytical rigour, the evolving thematic framework was then reviewed and discussed iteratively with two other research team members (X.H.W. and Y.Y.) until consensus was reached, ensuring the final themes accurately captured participants’ perspectives.

## Supplementary information

Supplementary information

## Data Availability

The datasets generated and/or analyzed during the current study are not publicly available due to ethical restrictions imposed by the University of Manchester during the ethics review process, which require that data must not be shared with any external individuals or organizations in accordance with the University’s data protection policy. However, data are available from the corresponding author on reasonable request.

## Code availability

No custom code was generated or analysed during the current study.
