# Supplementary material for: Feasibility of social media health education plus exercise in older adults with possible sarcopenia
Source: NPJ Digit Med. 2026 Apr 8;9:430. doi: 10.1038/s41746-026-02585-7 (PMC13234179; doi:10.1038/s41746-026-02585-7)
Supplement: Supplementary file 1 — Supplementary information [file 41746_2026_2585_MOESM1_ESM.pdf]

Supplementary materials

Supplementary Table 1

Prevalence of possible sarcopenia in older people according to different cross-sectional research

Researcher	Year	Country	Setting	Criteria	Sample	Age (years)	Prevalence (%)		
							Overall	Female	Male
Wearing J [1]	2020	Switzerland	Community	EWGSOP2	219	≥75	26.9%	26.3	28.0
Tsekoura M [2]	2021	Greece	Community	EWGSOP2	402	>60	25.4%	21.2	36.4
Paludo CDS [3]	2024	Brazil	Rural area	EWGSOP2	651	≥60	19.2%	14.3	23.8
Lim SK [4]	2022	South Korea	Community	AWGS2	431	≥65	23.7%	24.35	21.95
Pérez-Sousa MÁ [5]	2021	Colombia	Urban and rural area	EWGSOP2	5237	≥60	46.5	45.4	47.9
Ramirez E [6]	2022	Mexico	Community	EWGSOP2	1283	--	46.5	--	--
Chen Z [7]	2022	China	Community	AWGS2	4866	≥60	46.0	51.3	40.8
Yao J [8]	2022	China	Community	AWGS2	1082	≥60	48.10	44.48	52.79

Note: '--' means that the data was not obtained.

Supplementary Table 2

Prevalence of three categories of sarcopenia in older people according to different cross-sectional research

Researcher	Year	Country	Setting	Criteria	Sample	Age (years)	Prevalence (%)		
							Possible	Confirmed	Severe
Sobestiansky S [9]	2019	Sweden	Home and university hospital	EWGSOP2	354	85-89	73.0	20.0	1.8
Kim M [10]	2019	South Korea	Community	EWGSOP2	2099	70-84	23.5	18.4	3.9
Murphy CH [11]	2023	Ireland	Community	EWGSOP2	308	≥65	20.8	8.1	5.8
Guillamón-Escudero C [12]	2020	Spain	Community	EWGSOP2	202	≥65	18.8	3.0	4.5
Chew J [13]	2020	China	Community	EWGSOP2	200	≥50	21.0	14.5	1.5
Wu X [14]	2021	China	Community	AWGS2	6172	≥60	38.5	18.6	8.0
Wang Jm [15]	2022	China	Community	AWGS2	729	≥60	11.11	10.29	
Pang BWJ [16]	2020	Singapore	Community	AWGS2	297	>60	16.8	23.9	17.2

Supplementary References

1. Wearing, J., Konings, P., de Bie, R. A., Stokes, M. & de Bruin, E. D. Prevalence of probable sarcopenia in community-dwelling older Swiss people - a cross-sectional study. *BMC Geriatr* **20**, 307 (2020).
2. Tsekoura, M., Billis, E., Matzaroglou, C., Tsepis, E. & Gliatis, J. Prevalence of probable sarcopenia in community-dwelling older Greek people. *J Frailty Sarcopenia Falls* **6**, 204–208 (2021).
3. Paludo, C. D. S., Gonzalez, T. N., Soares, P. S. M. & Meucci, R. D. Prevalence and factors associated with probable sarcopenia in southern Brazil: a population-based study. *Rural Remote Health* **24**, 8711 (2024).
4. Lim, S. K. & Kong, S. Prevalence, physical characteristics, and fall risk in older adults with and without possible sarcopenia. *Aging Clin Exp Res* **34**, 1365–1371 (2022).
5. Pérez-Sousa, M. Á., Pozo-Cruz, J. D., Cano-Gutiérrez, C. A., Izquierdo, M. & Ramírez-Vélez, R. High Prevalence of Probable Sarcopenia in a Representative Sample From Colombia: Implications for Geriatrics in Latin America. *J Am Med Dir Assoc* **22**, 859-864.e1 (2021).
6. Ramirez, E., Salas, R., Bouzas, C., Pastor, R. & Tur, J. A. Comparison between Original and Reviewed Consensus of European Working Group on Sarcopenia in Older People: A Probabilistic Cross-Sectional Survey among Community-Dwelling Older People. *Gerontology* **68**, 869–876 (2022).
7. Chen, Z., Ho, M. & Chau, P. H. Prevalence, Incidence, and Associated Factors of Possible Sarcopenia in Community-Dwelling Chinese Older Adults: A Population-Based Longitudinal Study. *Front Med (Lausanne)* **8**, 769708 (2021).
8. Yao, J. *et al.* Prevalence of possible sarcopenia in community-dwelling older Chinese adults: a cross-sectional study. *BMJ Open* **12**, e067425 (2022).
9. Sobestiansky, S., Michaelsson, K. & Cederholm, T. Sarcopenia prevalence and associations with mortality and hospitalisation by various sarcopenia definitions in 85-89 year old community-dwelling men: a report from the ULSAM study. *BMC Geriatr* **19**, 318 (2019).
10. Kim, M. & Won, C. W. Prevalence of sarcopenia in community-dwelling older adults using the definition of the European Working Group on Sarcopenia in Older People 2: findings from the Korean Frailty and Aging Cohort Study. *Age Ageing* **48**, 910–916 (2019).
11. Murphy, C. H. *et al.* Prevalence and determinants of sarcopenia in community-dwelling older adults in Ireland. *Aging Clin Exp Res* **35**, 1651–1660 (2023).
12. Guillamón-Escudero, C., Diago-Galmés, A., Tenías-Burillo, J. M., Soriano, J. M. & Fernández-Garrido, J. J. Prevalence of Sarcopenia in Community-Dwelling Older Adults in Valencia, Spain. *Int J Environ Res Public Health* **17**, 9130 (2020).
13. Chew, J. *et al.* Muscle Strength Definitions Matter: Prevalence of Sarcopenia and Predictive Validity for Adverse Outcomes Using the European Working Group on Sarcopenia in Older People 2 (EWGSOP2) Criteria. *J Nutr Health Aging* **24**, 614–618 (2020).
14. Wu, X. *et al.* Sarcopenia prevalence and associated factors among older Chinese population: Findings from the China Health and Retirement Longitudinal Study. *PLoS One* **16**, e0247617 (2021).
15. Wang, J. *et al.* Prevalence and associated factors of possible sarcopenia and sarcopenia: findings from a Chinese community-dwelling old adults cross-sectional study. *BMC Geriatr* **22**, 592 (2022).
16. Pang, B. W. J. *et al.* Prevalence and Associated Factors of Sarcopenia in Singaporean Adults-The Yishun Study. *J Am Med Dir Assoc* **22**, 885.e1-885.e10 (2021).

Supplementary Note 1

Baseline characteristics of participants by study completion status

Participants were categorized into three groups based on their study engagement: completers (completed all outcome assessments and the final follow-up, n=26), follow-up completers without interview (completed the outcome assessment at T13 but not the optional qualitative interview, n=6), and early withdrawals (discontinued before the final assessment, n=3). Baseline characteristics for these groups are summarized in Supplementary Table 3 and 4. Descriptive comparisons suggested generally similar distributions across groups for demographic, health, and physical function variables. Where methodologically appropriate (i.e., between completers [n=26] and the follow-up completers without interview [n= 6]), Mann–Whitney U tests were performed and revealed no statistically significant differences in any baseline characteristic (all p > 0.05). However, these comparisons were severely underpowered due to the small size of the withdrawal subgroups, and non-significant results should not be interpreted as evidence of equivalence. Given the extremely limited sample size of the early withdrawal group (n=3), inferential statistics were not conducted for this subgroup; data are presented for descriptive purposes only. Overall, the available data do not suggest systematic baseline differences between those who completed the study and those who withdrew, though the ability to detect such differences was limited by the low number of withdrawals.

Supplementary Table 3

Categorical baseline characteristics of participants by study completion status [n(%)]

Characteristic		Completers (n=26)	Completed Follow-up, No Interview (n=6)	Early Withdrawals (n=3)	*U*	*p*
Age group	60-64	5 (19.2)	1 (16.7)	1 (33.3)	76.000	0.886
	65-69	21 (80.8)	5 (83.3)	2 (66.7)		
Gender	Female	22 (84.6)	3 (50.0)	3 (100.0)	51.000	0.069
Education	Primary school	8 (30.8)	3 (50.0)	0 (0.0)	69.000	0.645
	Junior high school	10 (38.5)	1 (16.7)	1 (33.3)		
	Senior high school	8 (30.8)	2 (33.3)	2 (66.7)		
Long-term condition	Hypertension	12 (46.2)	4 (66.7)	0 (0.0)	62.000	0.373
	Diabetes	5 (19.2)	3 (50.0)	0 (0.0)	54.000	0.123
	Hyperlipidaemia	5 (19.2)	1 (16.7)	0 (0.0)	76.000	0.886
	Coronary heart disease	1 (3.8)	0 (0.0)	0 (0.0)	75.000	0.631
	Lumbar disc herniation	1 (3.8)	0 (0.0)	1 (33.3)	75.000	0.631
	Arthritis	1 (3.8)	0 (0.0)	0 (0.0)	75.000	0.631

Medication	Yes	14 (53.8)	4 (44.7)	0 (0.0)	68.000	0.574
Significant health problems affecting the ability to undertake physical activity	Yes	0 (0.0)	0 (0.0)	0 (0.0)	78.000	1.000
Mobility aid	Yes	1 (16.7)	0 (0.0)	0 (0.0)	75.000	0.906

Note: Due to the very small sample sizes in the withdrawal subgroups (n=6 and n=3), formal statistical comparisons are not presented or are interpreted with extreme caution. Where methodologically appropriate (i.e., for comparisons between completers [n=26] and the largest withdrawal subgroup [n=6]), Mann-Whitney U tests were conducted and indicated no statistically significant differences in any baseline characteristic (all $p > 0.05$). However, these tests are severely underpowered. Data for the early withdrawal group (n=3) are presented for descriptive purposes only.

Supplementary Table 4

Continuous baseline characteristics of participants by study completion status (mean ± SD)

	Completers (n=26)	Completed Follow-up, No Interview (n=6)	Early Withdrawals (n=3)	*U*	*p*
Right handgrip strength, kg	16.46 ± 4.36	16.86 ± 6.58	19.88 ± 6.89	68.500	0.646
Left handgrip strength, kg	15.27 ± 4.65	17.41 ± 3.75	17.83 ± 6.67	45.000	0.111
4-metre walking speed, m/s	1.29 ± 0.17	1.25 ± 0.14	1.38 ± 0.26	65.000	0.530
Sit to stand time, s	8.12 ± 1.54	8.16 ± 1.51	8.12 ± 2.36	64.500	0.514
Skeletal muscle mass, kg	24.02 ± 5.41	26.77 ± 5.10	25.60 ± 3.64	51.500	0.201
Skeletal mass index, kg/m^2	7.82 ± 1.34	8.55 ± 1.53	8.63 ± 0.49	44.500	0.105
Body mass index, kg/m^2	23.32 ± 2.75	24.01 ± 2.38	25.92 ± 2.51	74.000	0.847
Mini-Nutritional Assessment Short Form	13.35 ± 0.98	13.50 ± 0.55	14.00 ± 0.00	76.500	0.934

Note: Due to the very small sample sizes in the withdrawal subgroups (n=6 and n=3), formal statistical comparisons are not presented or are interpreted with extreme caution. Where methodologically appropriate (i.e., for comparisons between completers [n=26] and the largest withdrawal subgroup [n=6]), Mann-Whitney U tests were conducted and indicated no statistically significant differences in any baseline characteristic (all $p > 0.05$). However, these tests are severely underpowered. Data for the early withdrawal group (n=3) are presented for descriptive purposes only.

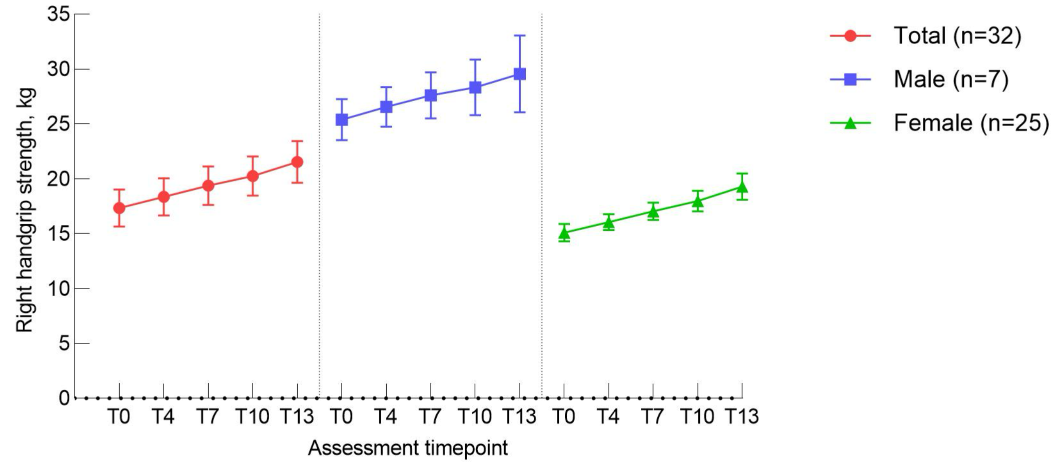

(a) Right handgrip strength, kg

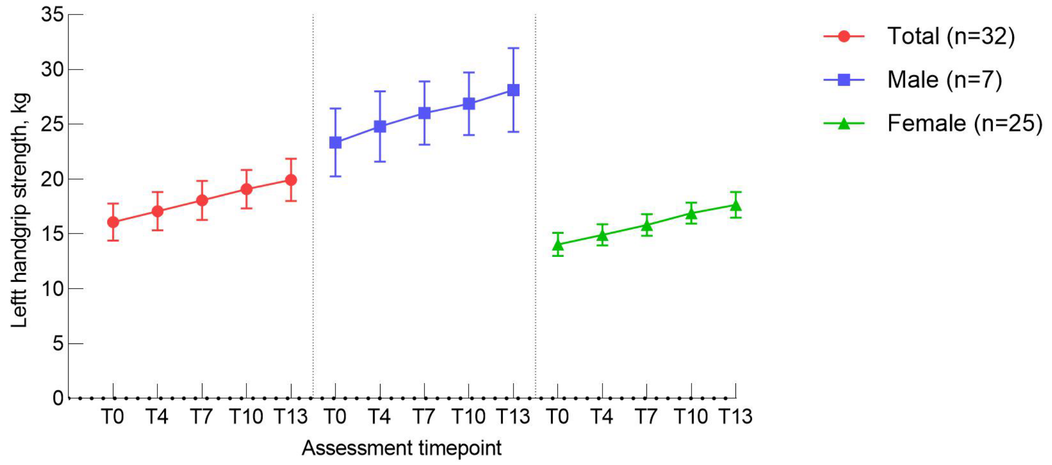

(b) Left handgrip strength, kg

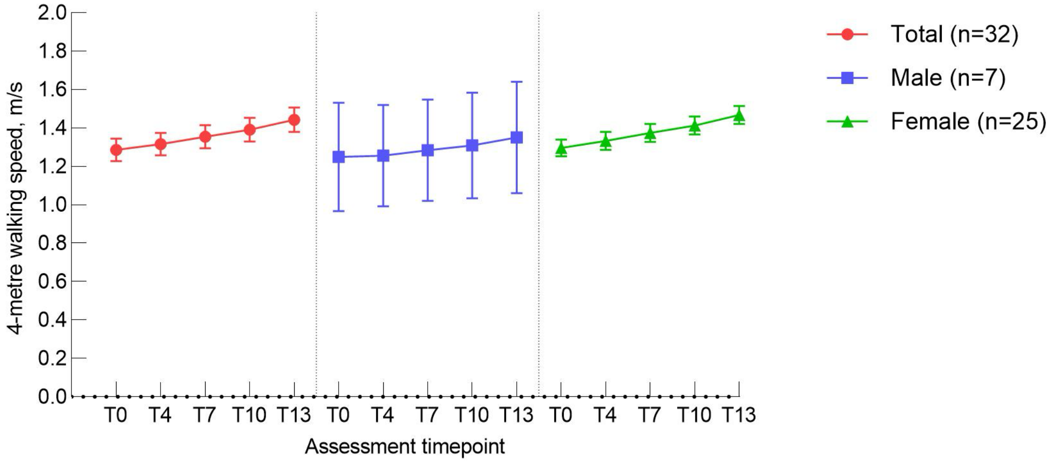

(c) 4-metre walking speed, m/s

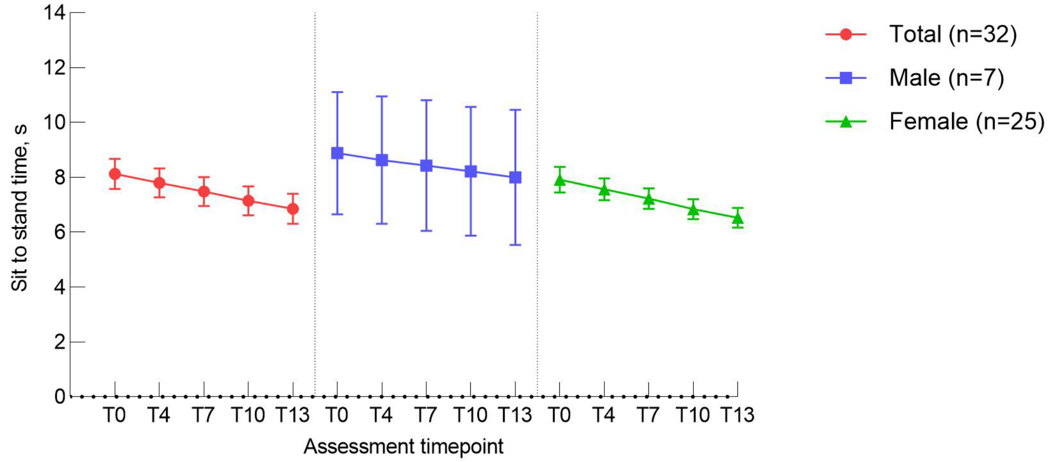

(d) Sit to stand time, s

Supplementary Figure 1. Trends in functional capacity indicators from baseline to week 13

Note: Data points represent mean values; error bars indicate 95% confidence intervals.

T_0 = Baseline; T_1= Week 1 (health education); T_4= Week 4 (exercise); T_7 = Week 7 (exercise); T_{10} = Week 10 (follow-up); T_{13} = Week 13 (follow-up).

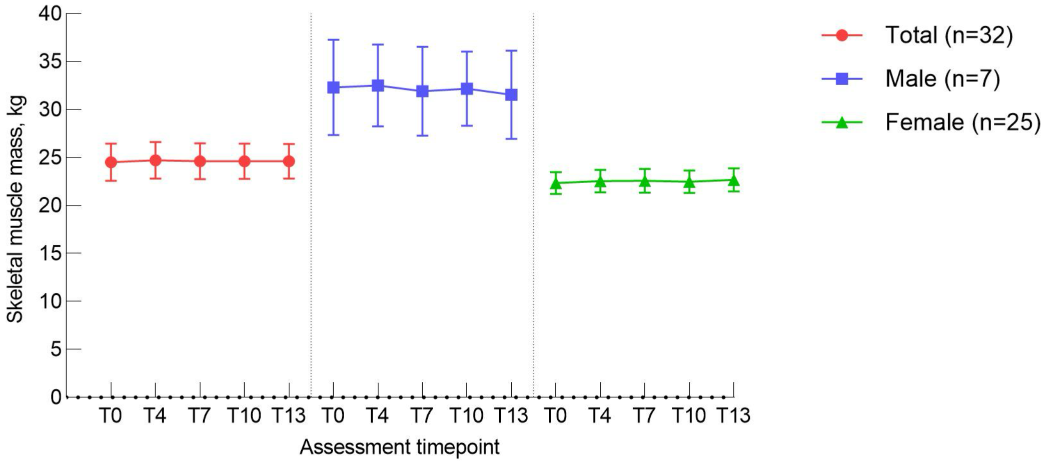

(a) Skeletal muscle mass, kg

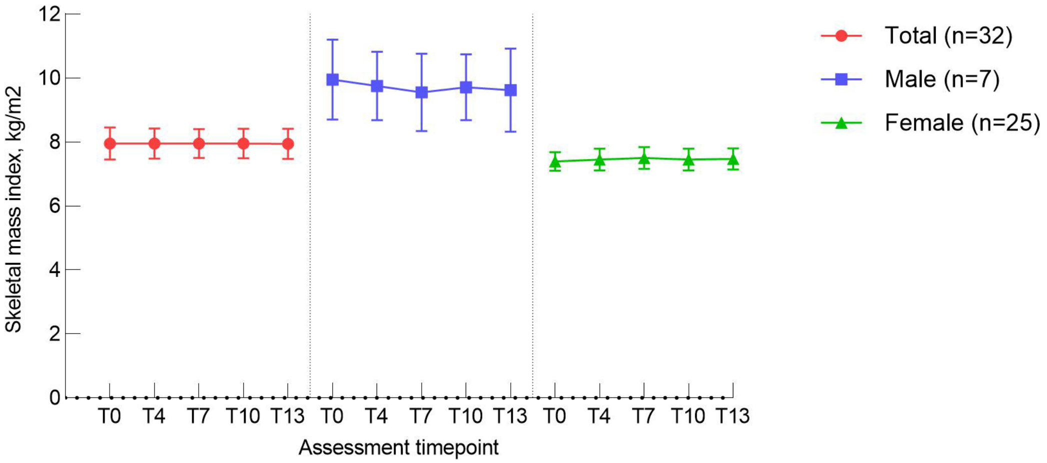

(b) Skeletal mass index, kg/m^2

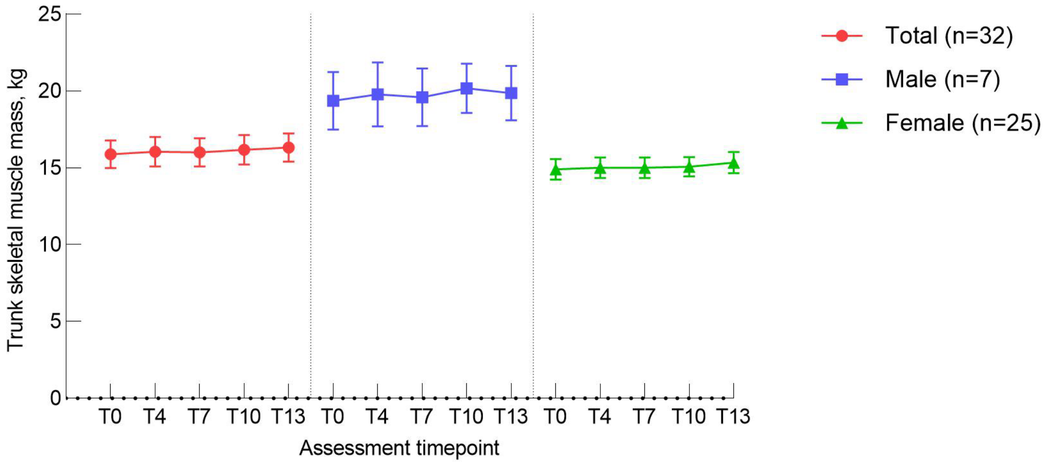

(c) Trunk skeletal muscle mass, kg

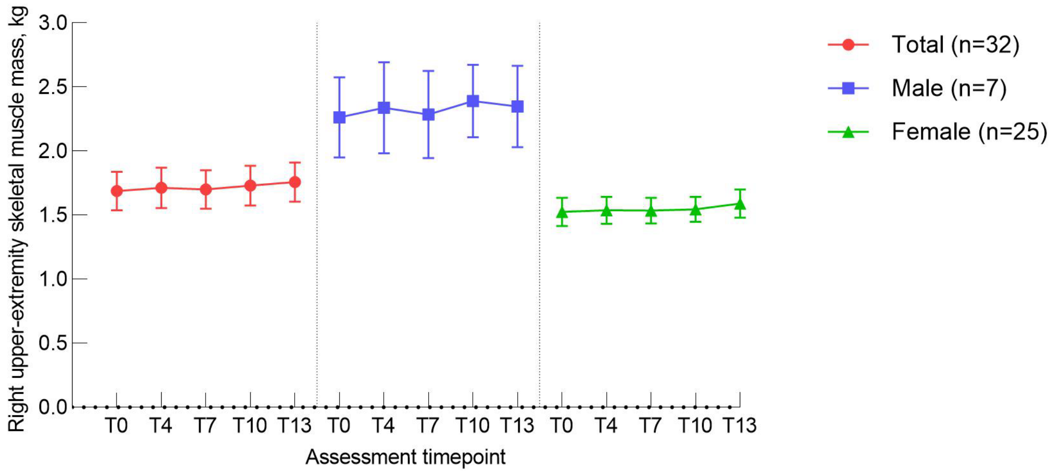

(d) Right upper-extremity skeletal muscle mass, kg

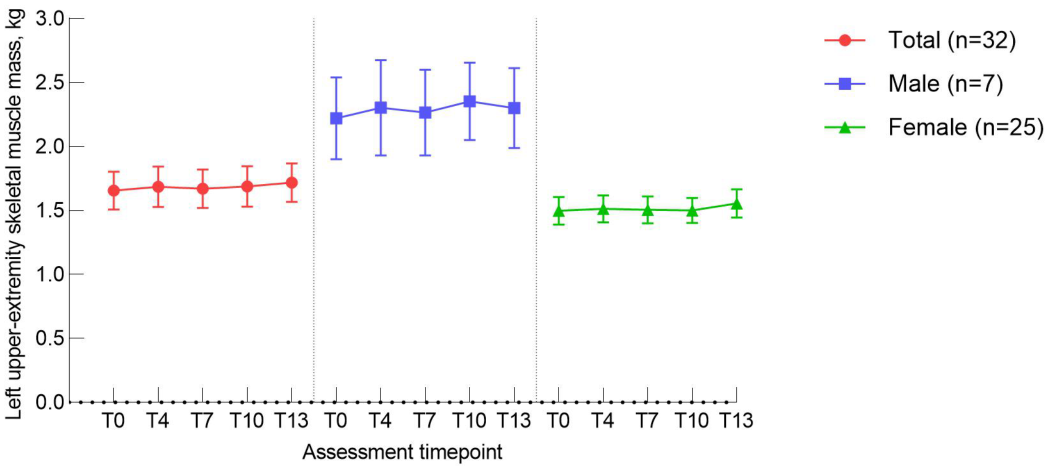

(e) Left upper-extremity skeletal muscle mass, kg

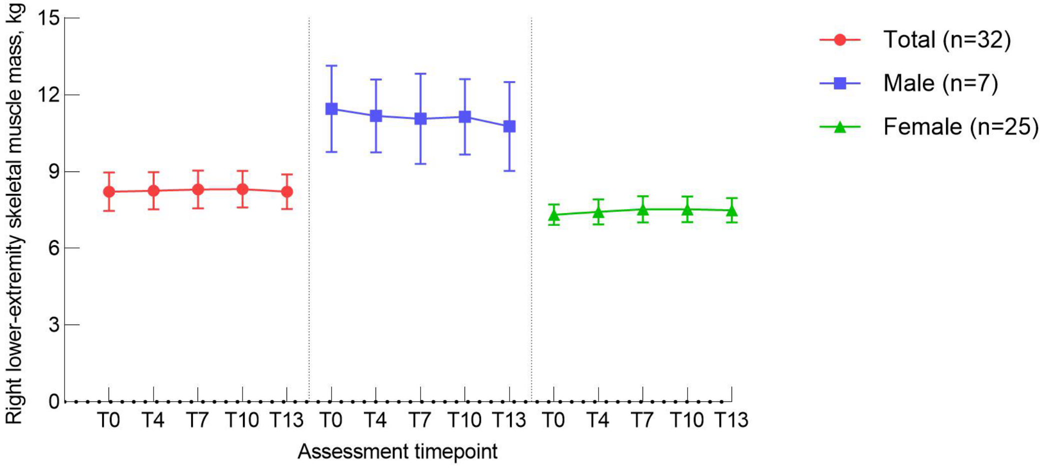

(f) Right lower-extremity skeletal muscle mass, kg

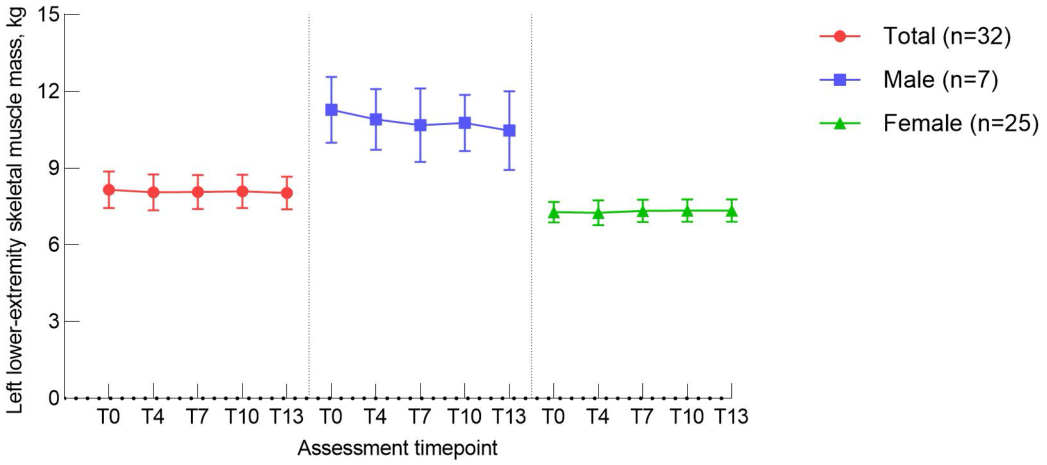

(g) Left lower-extremity skeletal muscle mass, kg

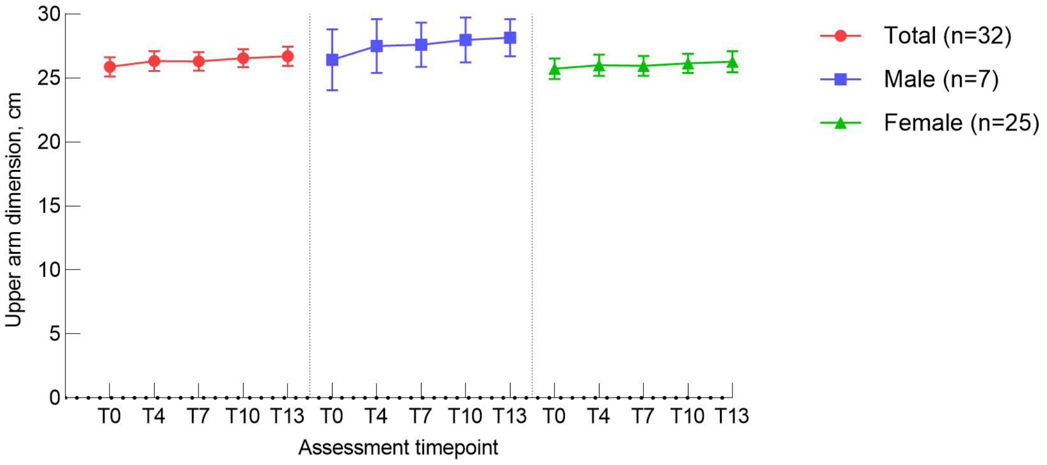

(h) Upper arm dimension, cm

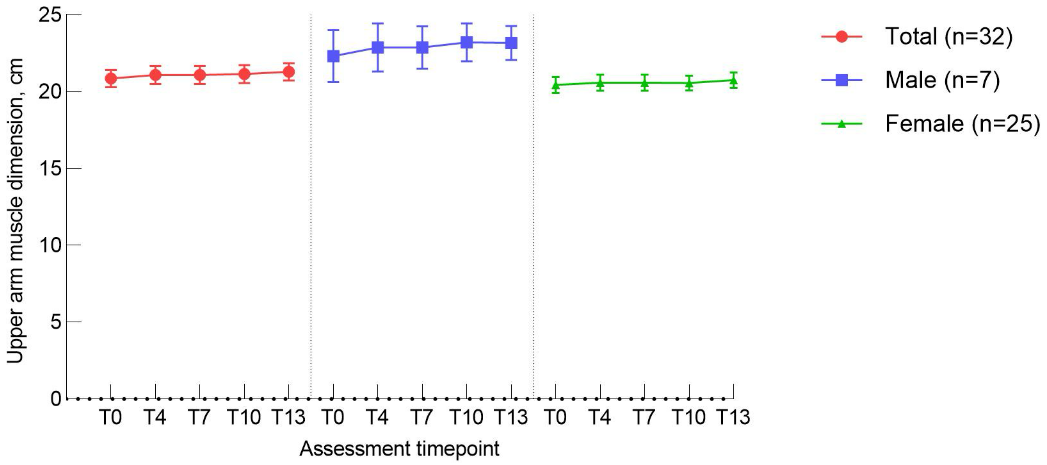

(i) Upper arm muscle dimension, cm

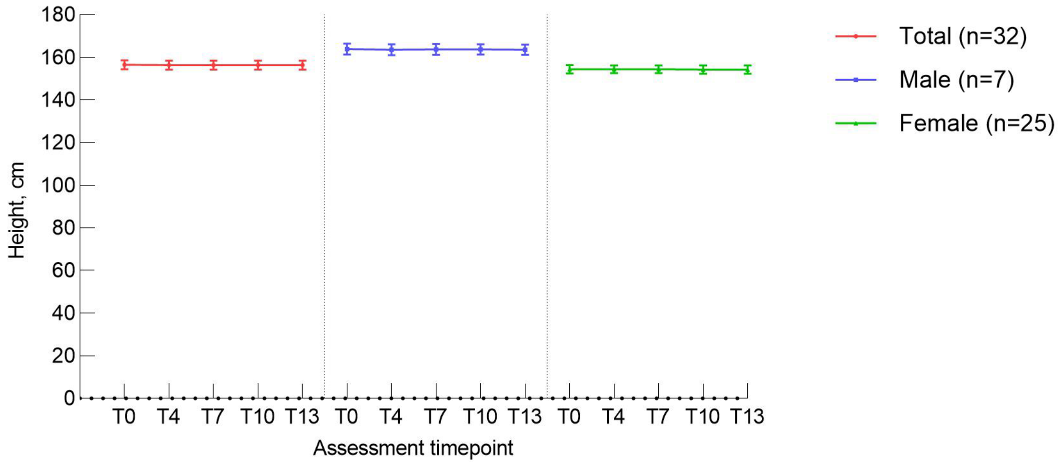

(j) Height, cm

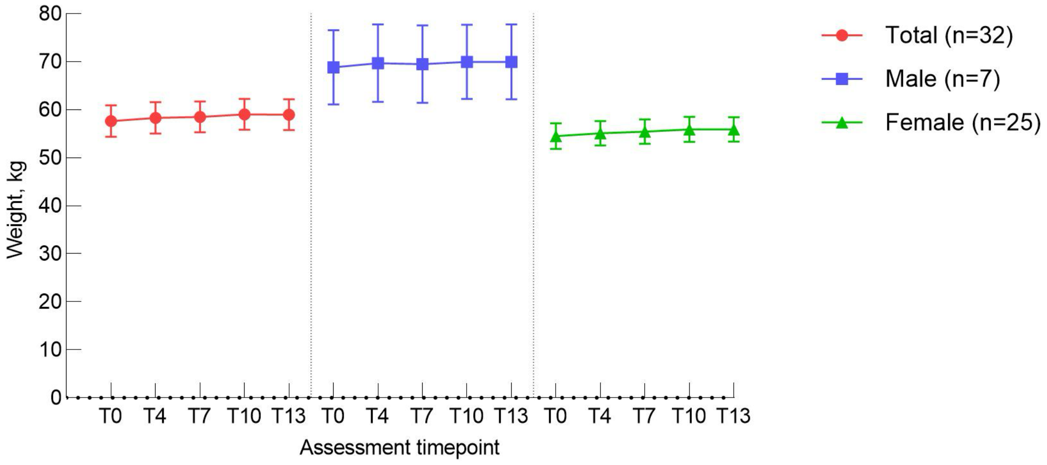

(k) Weight, kg

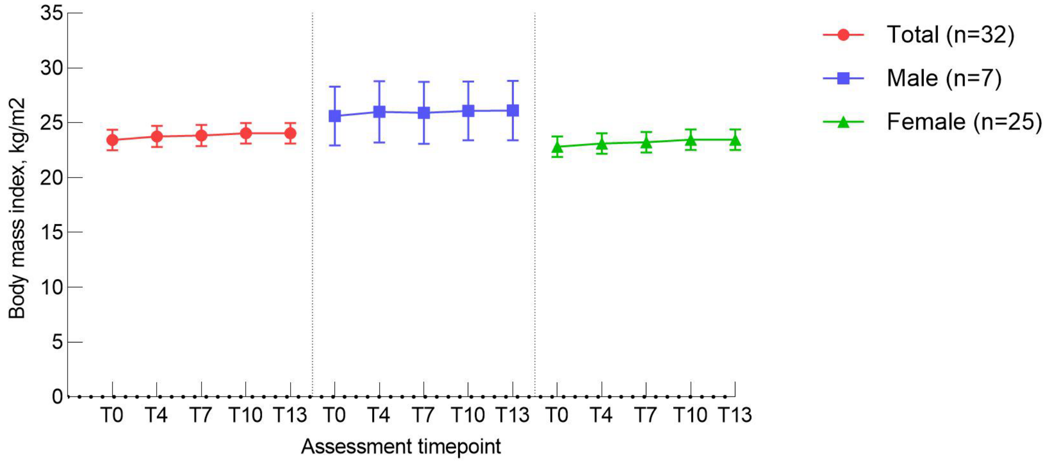

(l) Body mass index, kg/m^2

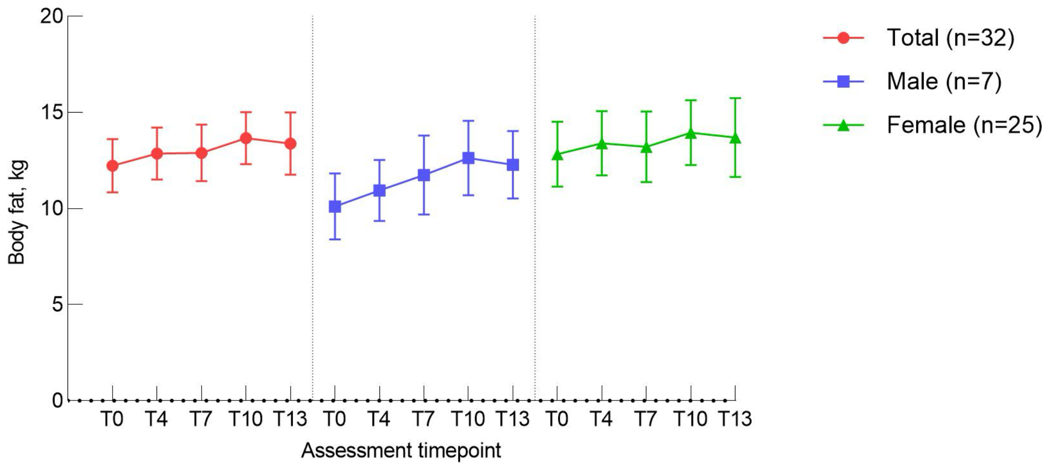

(m) Body fat, kg

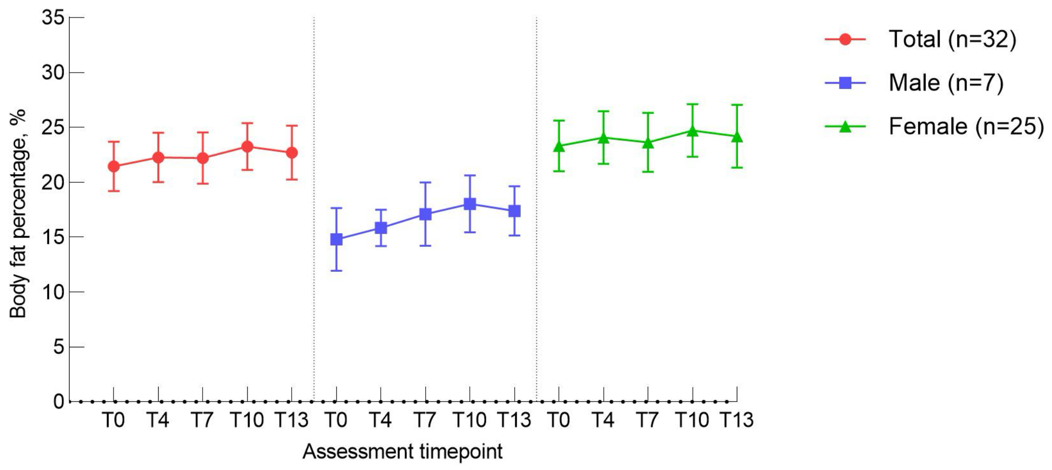

(n) Body fat percentage, %

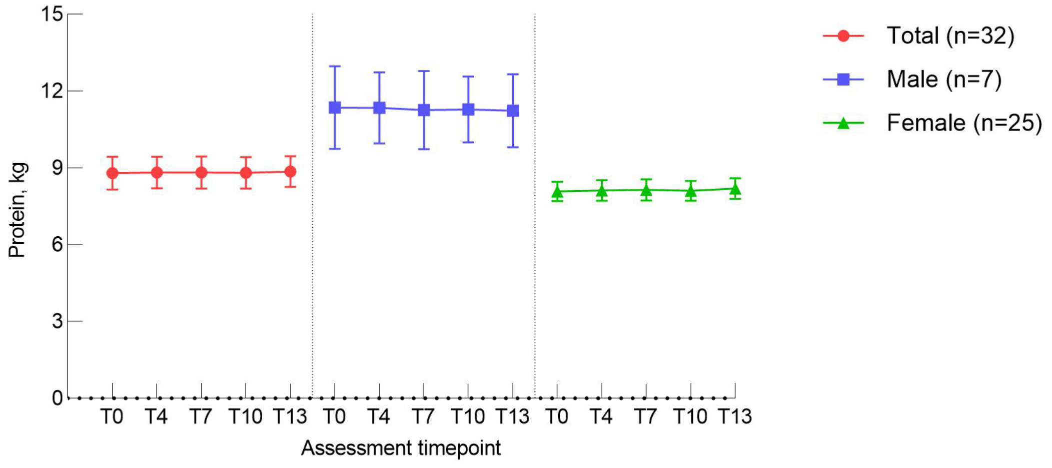

(o) Protein, kg

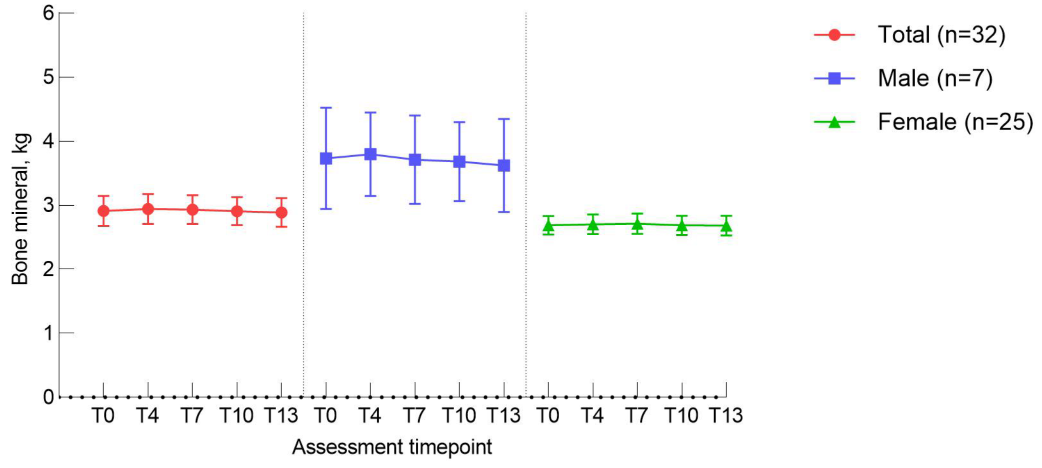

(p) Bone mineral, kg

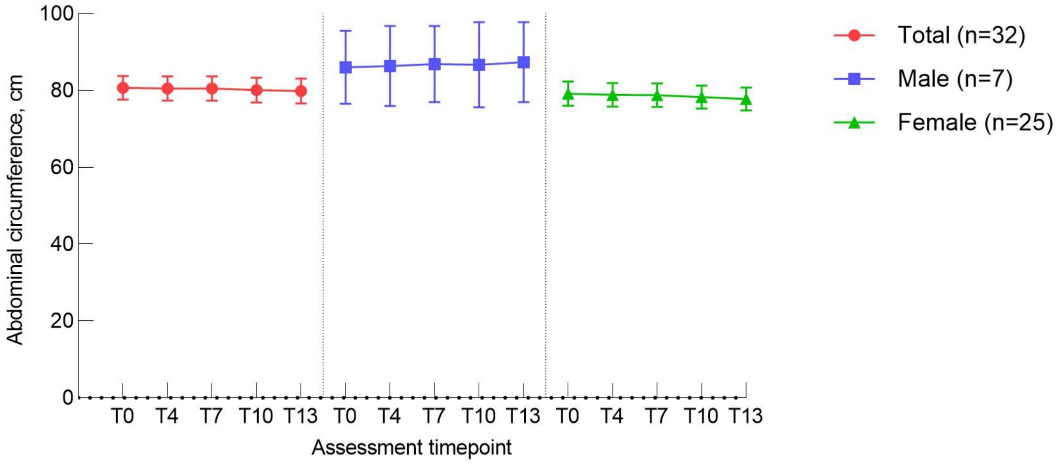

(q) Abdominal circumference, cm

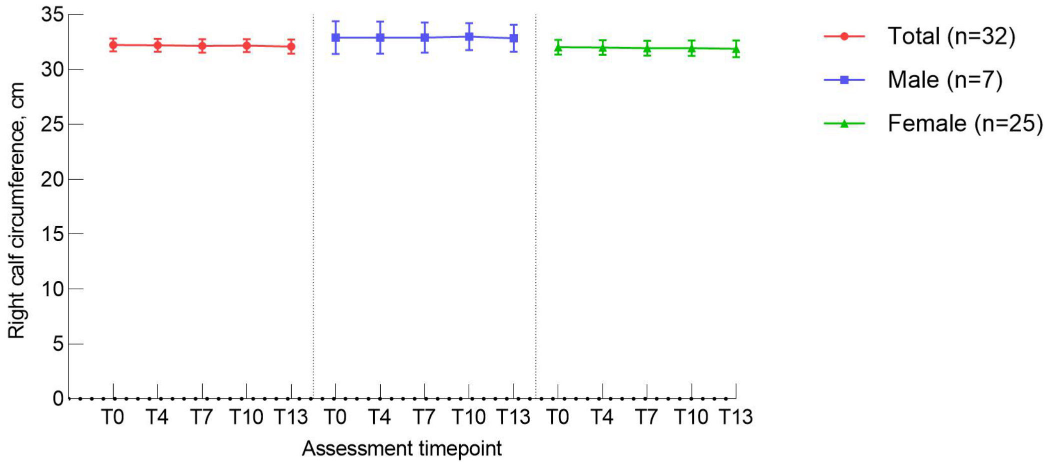

(r) Right calf circumference, cm

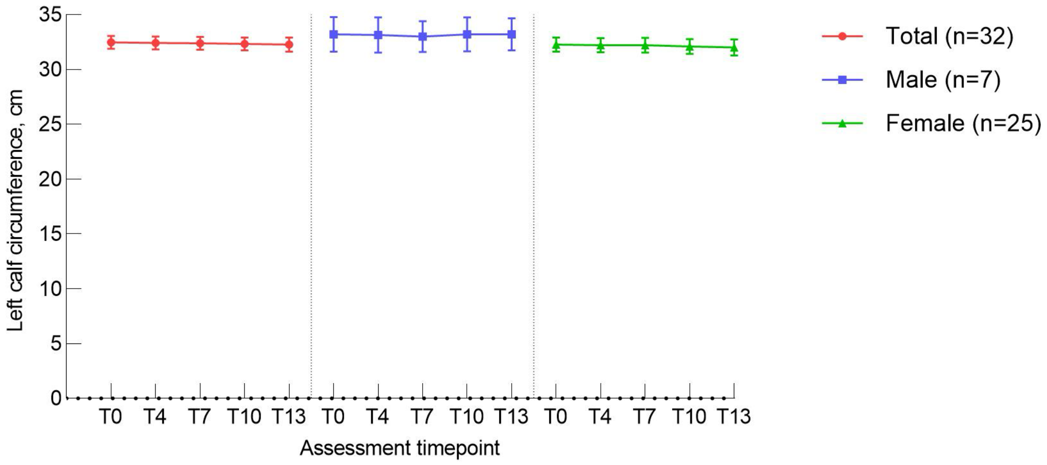

(s) Left calf circumference, cm

Supplementary Figure 2. Trends in body composition indicators from baseline to week 13

Note: Data points represent mean values; error bars indicate 95% confidence intervals.

T_0 = Baseline; T_1= Week 1 (health education); T_4= Week 4 (exercise); T_7 = Week 7 (exercise); T_{10} = Week 10 (follow-up); T_{13} = Week 13 (follow-up).

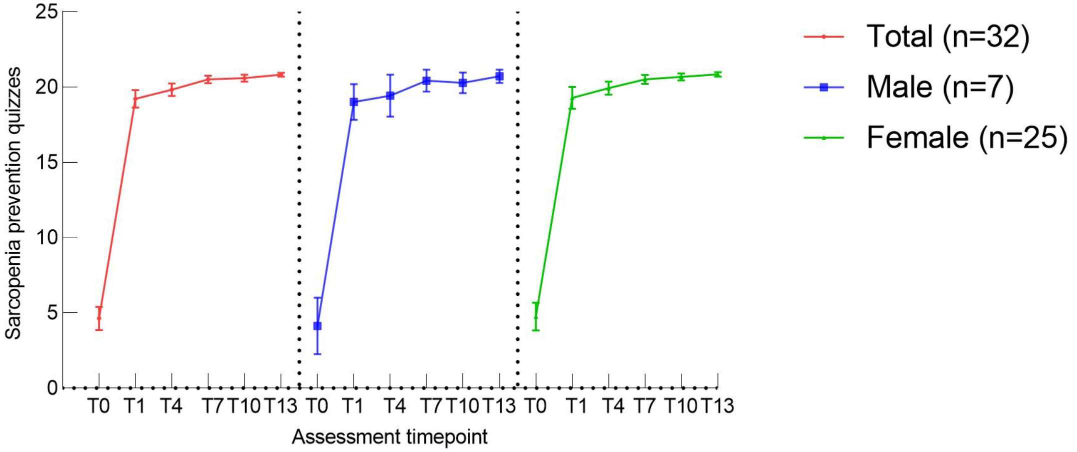

(a) Sarcopenia prevention quizzes score, points

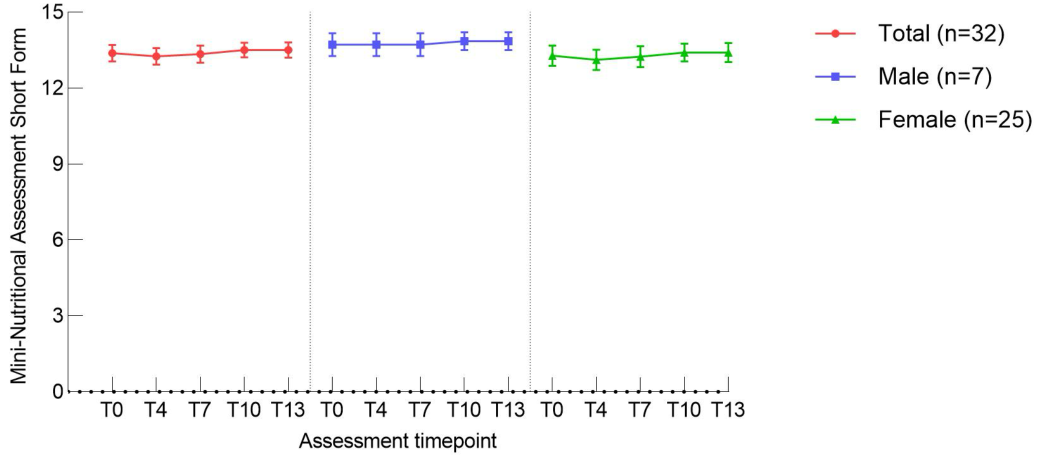

(b) Mini-Nutritional Assessment Short Form, points

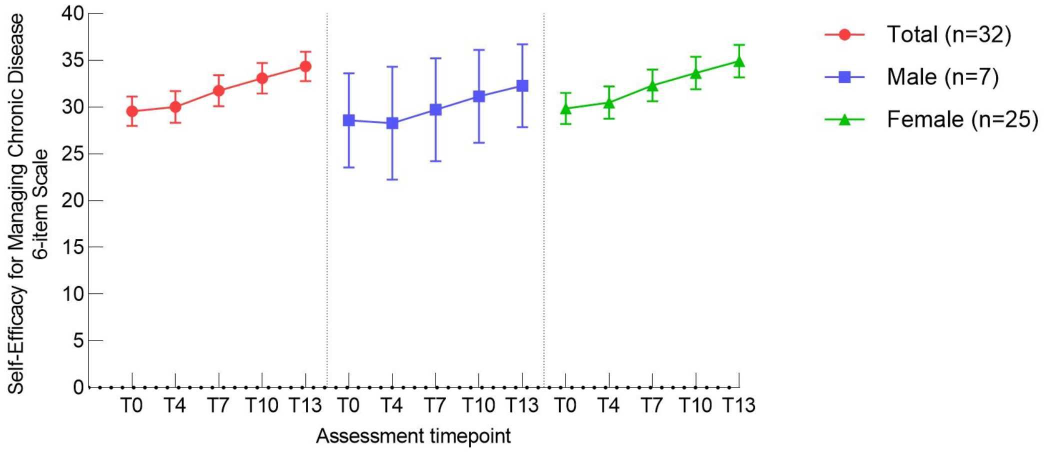

(c) Self-Efficacy for Managing Chronic Disease 6-item Scale, points

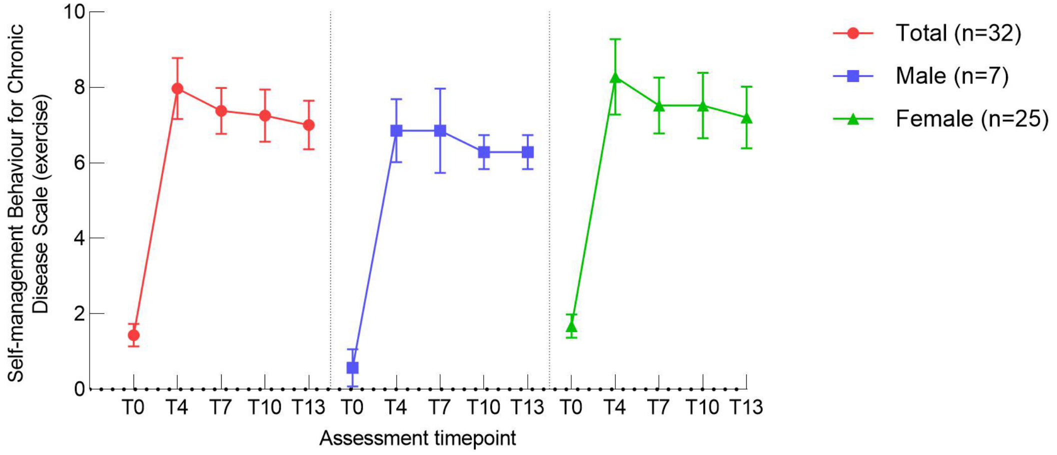

(d) Self-management Behaviour for Chronic Disease Scale (exercise), points

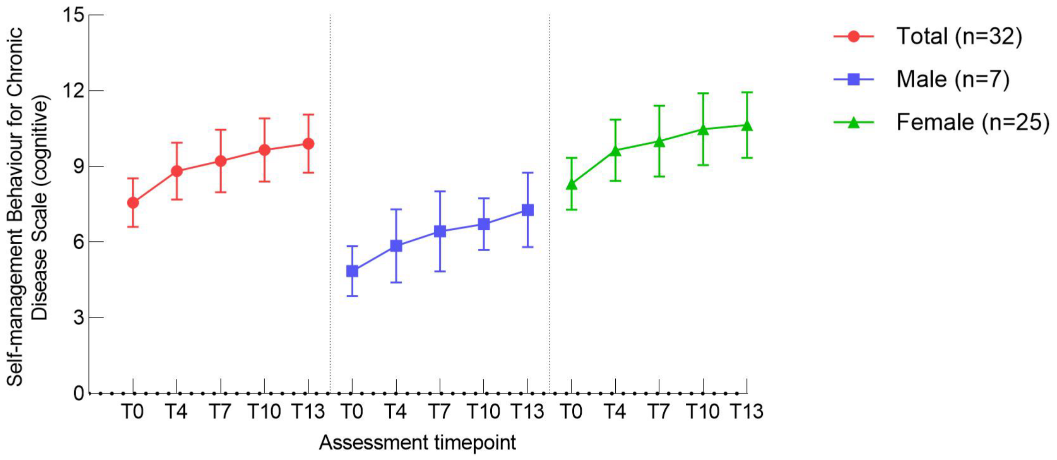

(e) Self-management Behaviour for Chronic Disease Scale (cognitive), points

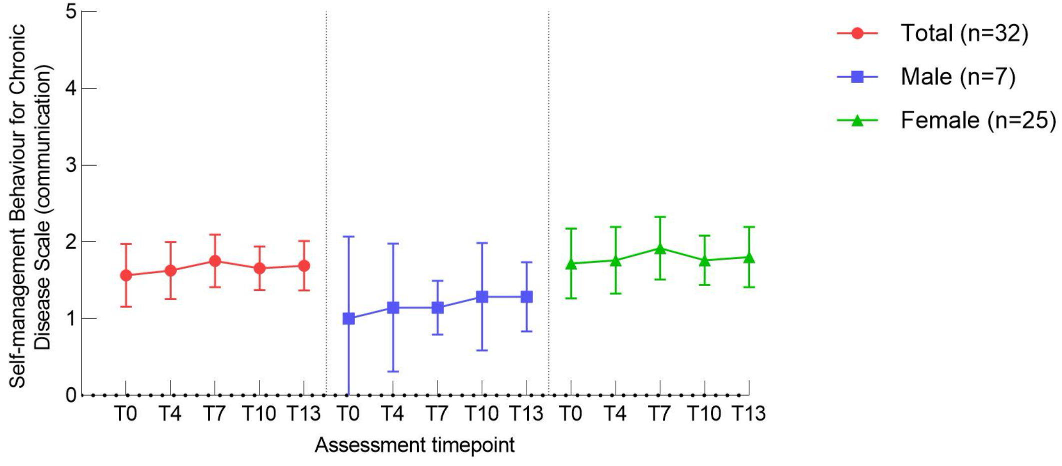

(f) Self-management Behaviour for Chronic Disease Scale (communication) , points

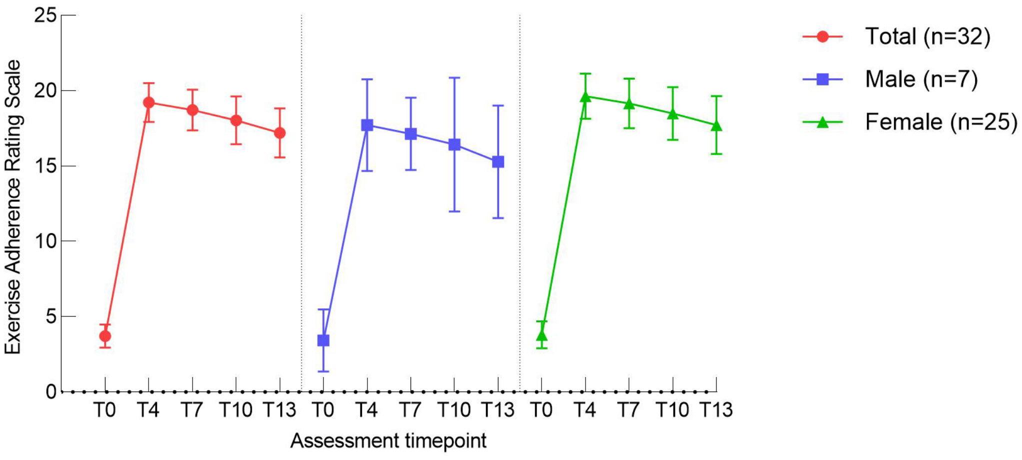

(g) Exercise Adherence Rating Scale, points

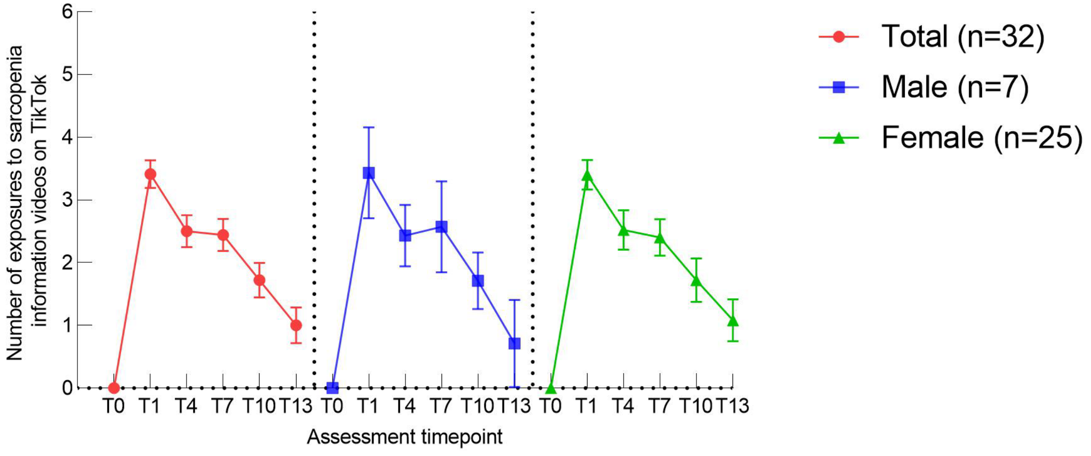

(h) Number of exposures to sarcopenia information videos on TikTok, cnt

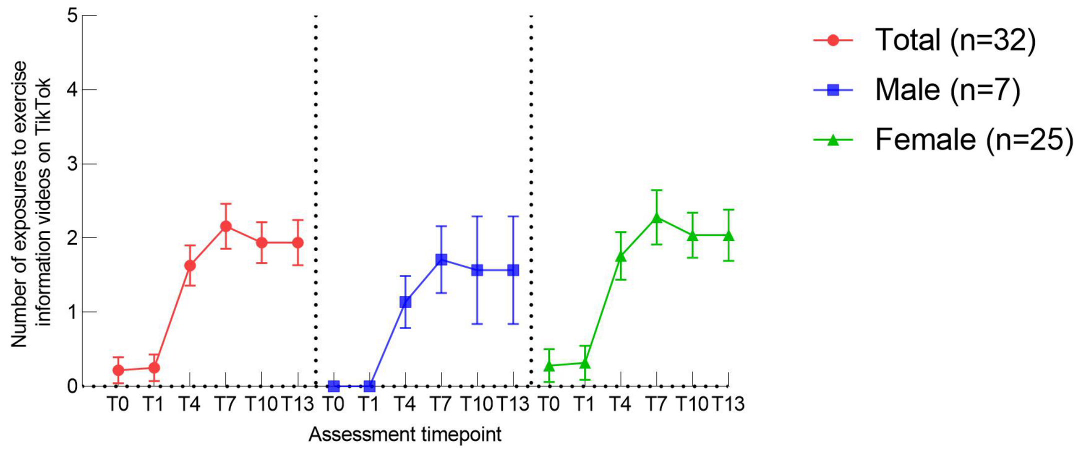

(i) Number of exposures to exercise information videos on TikTok, cnt

Supplementary Figure 3. Trends in knowledge, psychosocial factors, and behavioural indicators from baseline to week 13

Note: Data points represent mean values; error bars indicate 95% confidence intervals.

T_0 = Baseline; T_1= Week 1 (health education); T_4= Week 4 (exercise); T_7 = Week 7 (exercise); T_{10} = Week 10 (follow-up); T_{13} = Week 13 (follow-up).

Supplementary Note 2

Discourse characteristics of the interviewees

· ***Theme 1: The comprehensive evaluation for this research***

1) Study procedures

All 26 participants expressed satisfaction with their involvement in the study and reported feeling relaxed and content throughout its period.

P12: "There was no pressure throughout the study, and it was quite comfortable for me to participate in the entire procedure. Your work was also quite professional, and I was really pleased throughout the process. I hope that if you return in the future, I can still follow you to learn about sarcopenia prevention, but I am scared you will not come."
P17: "My opinion of the entire study process is very positive. If I must award grades, I believe I can give full marks. None of the aspects were complex or demanding, and the entire process was simple."
P35: "The entire study is excellent, especially for older people. There were no complicated or time-consuming parts to the procedure; it was really cheerful and relaxed, with no pressure."

2) Data collection and measurement

The 26 participants concurred that the data collecting, and measurement process was acceptable, with no reported stress or discomfort.

P9: "There was no unpleasant or stressful element of the study, no pressure throughout the measurement process, the procedure was pretty simple, and the overall quality was good."
P13: "The entire measurement process was not tough or demanding, and this research work was not difficult. Overall participation was still easy."
P33: "The entire measurement process was acceptable. There was no pressure or discomfort, and the participation was comfortable and relaxing. There was nothing to improve."

3) Researcher

14 (53.85%) participants actively mentioned and expressed favorable opinions towards the PI, which may have contributed to the participants' willingness to stick with the study.

P2: "The researcher was extremely patient and kind to older people."

P17: “As a researcher, I also approved you. Your attitude was excellent. You were also a part of what motivates me to continue with this project.”
P21: “I felt you were very patient. Your work was also quite serious and responsible, so I was willing to join.”

· ***Theme 2: The personal experience of the intervention***

1) Health education

Using the Likert-5 score, just one participant provided a satisfactory evaluation of the health education strategy, while the remaining 25 participants rated it as very satisfactory. The evaluation of health education strategy was categorised in four aspects: health education content, duration, frequency, and delivery tool.

① Health education content: All participants found the health education information easy to understand, and they benefited from the knowledge of sarcopenia prevention to varying degrees.

P5: “I had no idea about sarcopenia before, but thanks to health education, I now understand that I should take proactive steps to prevent it. All topics of health education are understandable. There are no tough parts, and I can follow all your lectures.”
P9: “Sarcopenia preventive knowledge was extremely useful. After learning, I became more cautious about my eating habit and exercise, which was good for my own health. I comprehended all the content of health information, and there was nothing that I did not grasp, and there was also not extremely complicated or tough area that I could not follow.”
P12: “My feeling is that the knowledge you taught us was very useful, for example, after learning your videos, I felt that at least I realized that there was such a disease in our life, because before you came, I did not understand this disease at all. Now I know that there is such a muscle disease, so I feel that the health education is valuable. Another is having the idea of exercise after learning. I did not like to exercise before. I often sited and did not move much. After learning your project, it turns out that long-term sedentary behaviour leads to what you call sarcopenia, so I feel that I need to exercise to prevent the occurrence of sarcopenia. In general, I feel that what you taught us is still very useful.”
P17: “If I hadn't learned the knowledge from you, I would have refused to consume eggs and milk since I dislike them. However, after participating in your project and watching these health education videos, I started insisting on eating an egg and drinking a glass of milk every day. I normally drink milk at 10:00 a.m. and eat an egg each morning.”

P25: "I feel that I now have a better grasp of this disease, and that preventing sarcopenia must be an important thing for me. Later in life, if you are unaware of sarcopenia, it might lead to a variety of additional problems. After completing the health education course, I will prioritize the prevention of sarcopenia in my subjective consciousness, and I am willing to exercise on my subjective purpose, as I have gained new knowledge from this project. However, I can only give a satisfactory rating but not very satisfactory for the content of health education. The reason is that I hope you will continue to add new knowledge in the future, so that I can learn constantly. Otherwise, if you do not give lectures in the future, I may not take it so seriously."

② Health education duration: 23 (88.46%) participants believed 4-6 minutes per video was appropriate, but three participants thought it could be extended a bit, as they wished to learn more information regarding sarcopenia prevention.

P5: "The duration of the health education video can be increased slightly, as can the content, in the hopes of better understanding the progression of sarcopenia."
P12: "I believe each video length of 4-6 minutes is acceptable. If the time is too long, older people will lose attention, for example, I have grandchildren at home to care for, and I do not have much time to watch a video, so this time is quite suitable for me. I think these videos are more refined, and there is less nonsense, and I feel more useful."
P21: "The videos are 4-6 minutes long and can be extended slightly to provide more information regarding sarcopenia; ideally, it would be better if you can update them each year."
P25: "The video lasting 4-6 minutes is acceptable, as the people get older, their brains are less able to tolerate extended videos. I feel like I can only do it for so long. The other is that I must do some chores at home, and I can only accept so much without losing tolerance."
P31: "I believe the duration of the health education video can be increased since I want to learn more about sarcopenia prevention, but based on everyone's opinion, it is not sensible for you to make it longer on my own advice."

③ Health education frequency: Participants exhibited variability in the frequency of video viewership during the initial week. Twelve (46.15%) participants reported viewing 1-2 videos simultaneously, five (19.23%) indicated they preferred to watch all videos in one time, while the remaining nine (34.62%) participants stated that their viewing quantity was contingent upon their mood, however they would complete all videos within the first week.

P3: "The number of health education videos I watched at one time was not sure, sometimes two, sometimes a few more, depending on the situation and mood of the day."
P6: "I generally watch 1-2 videos at a time, my habit of watching videos was not very fixed, completely depended about the day. I looked at it when I really wanted to."
P8: "I used to view all the health education videos once. I could understand each topic, but I don't have excellent memory and would forget the details."
P17: "I normally watched 1-2 videos at a time, and occasionally I just put my phone next to me to listen. I would limit the amount of time I spent watching videos because my eyes would tire.
P25: "I watched all the videos at once, from what is sarcopenia to how to prevent sarcopenia. Sometimes I also checked up additional information on the Internet when I didn't understand anything, just to ensure that it's correct and that it's beneficial for me."

④ Delivery tool of health education: All participants deemed the acquisition of health education knowledge via TikTok to be acceptable and user-friendly.

P3: "The TikTok platform is easy to use, convenient, and appropriate for older people. The watching time is quite flexible, and I can watch at any time when I want."
P5: "The TikTok platform for learning knowledge is acceptable and simple to use. I normally play on TikTok since it is easier."
P10: "The TikTok platform is ideal for learning because it is simple to master. I enjoy watching TikTok. I do not play cards, sing, or dance, but I like viewing videos on TikTok, so I am more familiar with this platform.
P27: "It is beneficial to learn health education knowledge on the TikTok platform, as it is easier and more accessible. I hadn't played TikTok much before, but your initiative had taught me how to view videos on TikTok, which is quite intriguing."
P31: "The TikTok platform for learning knowledge is quite convenient, with nothing particularly challenging. After training, it is simple to enter. This platform is not complicated, and it is quite easy to accept by older population."

2) Exercise intervention

Using the Likert-5 score, all participants expressed very satisfaction with the overall assessment of the exercise strategy. The evaluation can be distilled into five aspects: exercise content, duration, frequency, promotional film, and delivery tool.

① Exercise content: All participants deemed the exercise content satisfactory and the movement design adequate. Using water bottles for resistance exercise was considered

novel by the participants. Besides, all participants reported no adverse reactions or discomfort during the exercise.

P12: "It's simple to exercise with water bottles. I don't have dumbbells at home, and I didn't realize the water bottles could be used for exercising before. So, after participating in your program, I believe that exercise is quite convenient. I will keep the large, excellent water bottles at home. The way of exercising with water bottles is excellent, and it is especially useful for older people in the family. After watching these exercise videos, I want to exercise. I think I like it."
P13: "The exercise content are ideal for me. All the exercises are simple and easy to understand, and I can master them from start to finish.
P21: "The exercise content is excellent. I have done these exercises when I wake up since they are more in line with my daily activities. They are simple to accept and are not difficult for me."
P27: "The exercise content is excellent, and the movement design is extensive. The movement difficulty is moderate. Nothing is unsatisfactory, and I can adapt very well."
P30: "I enjoy your exercise program, and my favourite is resistance exercise. I can do all the movements. Although I can't do some movements for too long, I can follow through. There are no challenging parts, and all are acceptable."
P31: "The resistance exercise with water bottle is incredibly convenient, and I've never participated in similar exercise before. I can now insist on exercise, even if you do not continue this project. Anyhow, now see the water bottles, I want to use them for resistance training."
P34: "The feeling of exercise with water bottles is like dumbbells. I had fun, and I exercised at home. My son-in-law and my husband both followed me to do the exercise. My son-in-law said, "I am in the gym, this is the same as dumbbells". I said, "Then I don't like dumbbells, I like water bottles." I think the dumbbell is the fixed weight, while I can control the weight of the water bottles freely. If I want to be light, I can dump a little water. Before joining this project, I did not think that water bottles can also be used for exercise."

② Exercise duration: 22 (84.62%) participants expressed that an exercise duration of 25 to 30 minutes was suitable, while the remaining 4 (15.38%) individuals deemed it acceptable to extend a little bit of the duration.

P6: "The exercise duration of 25-30min is adequate. It is not excessively long, otherwise individuals do not have time to engage. Anything longer will make me lose patience."
P8: "Exercise duration now is suitable, if too long, it can be difficult. They may be

able to lengthen some for those with good health. As for us in poor health, exercise must not be too lengthy, or we will be unable to adhere to it."
P12: "25 to 30 minutes is ideal. The duration cannot be too long, as many older people no longer live alone, and most of them must care for their families. Their children are working, and they must care for grandchildren. If the duration is longer, such as 40-50 minutes, family members sometimes come to you for something and interrupt your exercise. As a result, it will be very difficult for you to complete a complete exercise, so I believe 25-30 minutes is an adequate duration."
P21: "A 25–30-minute exercise is fine, but a little longer, with up to 40-50 minutes, is also acceptable for me."
P32: "The exercise duration of 25-30 minutes is acceptable. A bit longer is also fine, and I believe 45 minutes is sufficient."

③ Exercise frequency: All participants deemed a frequency of doing exercise at least three times weekly to be entirely attainable.

P3: "Three times a week is fine. I started doing it virtually every day, and then I did it every other day for a while. There is no time or place, depending on the situation. It could be in the morning or evening, in senior centre or at home."
P12: "Three times a week is a good frequency, and I believe everyone should be able to do it. I normally have one day exercise and one day rest, but if I'm not too busy, I can add another one or two times. I perform five or six exercises a week at most, and three or four times if I'm busy. So, I believe three times is totally okay."
P15: "The frequency of three times a week is acceptable, sometimes I will increase the frequency, depending on the situation. There was time to increase once or twice, to four or five times a week. Sometimes I am busy, however three times is basically no problem."
P17: "A frequency of three times a week is suitable. When I'm busy, I exercise every other day, so that's three or four times a week. When I'm free, I can do it every day."
P25: "I can do exercise around three times per week. My habit is that I like to participate in exercise with others and am lazy when doing it alone at home. Three times is fine if we all do it together. If it is too little like once a week, I don't think it's working."

④ Promotional films about exercise: All participants agreed that the promotional films of resistance exercise for sarcopenia prevention contributed to increase the awareness of sarcopenia prevention and improve the motivation for exercise.

P2: "After watching the promotional films, I was particularly concerned about spending the day in bed or in a wheelchair, so I now prioritize continuing to exercise,

and my body feels lot better after exercise. Older people have a difficult time recovering once they are in bed, so prevention should be the priority. I will now take the initiative to exercise every day, as I fear the negative consequences. These promotional films are relatively realistic and have the effect of reminding me to exercise. I'm now concerned that young people, like my daughter, who only sleep except for work, are sedentary all day. Their bodies are often uncomfortable, and they'll be less healthy than older people like us who exercise routinely."

P3: "The promotional films are excellent. After watching it, I feel it is much more helpful to older adults. I think they will pay more attention to sarcopenia prevention. It is beneficial to raise people's awareness of exercising."

P9: "Watching promotional films helped to raise awareness of sarcopenia prevention. Older people should continue to exercise, which is essential for maintaining good health. Otherwise, once older adults are in a wheelchair or bed, their quality of life suffers, both physically and mentally. This is detrimental to their children. It is preferable to exercise if I do not burden my children. It is critical to prevent sarcopenia and exercise more and lessen the stress on the family."

P12: "After watching the promotional films, I believe that exercise is vital. If older people do not exercise now, they will have difficulty recovering when they lie in bed. Prevention is extremely important. I will continue to exercise and not be a burden to my family. Because the moment you lie in bed, you need a caregiver. The salary of the caregiver is very expensive, which will cause a great burden to the family. I'd rather choose free exercise without spending money, and I also pay attention to my nutrition, to prevent disease. Why do we have to wait until it is too late to consider the need for self-care? So, I believe it is critical for our generation to understand prevention."

P17: "After seeing the promotional films, I realized that the older I am, the healthier I want to be. I do not want anything else, and I just want to be healthier. It's critical to insist on exercising. The films raised my awareness of sarcopenia prevention."

P25: "I feel important to focus on sarcopenia prevention after watching the promotional films. There are numerous heavy objects at home that may be used for resistance training, and you can do it at any time, but the challenge is sticking to it. Many problems, if not paid attention to now, you will gradually suffer from sarcopenia as you aged, which will lead to a slew of other issues in the future. The first step is to perform these exercises, particularly resistance exercises. The second is nutrition, which strengthens items such as protein. If you follow these preventive actions, you will make progress for yourself while also reducing the stress on your family."

⑤ Delivery tool of exercise: All participants deemed the usage of TikTok for exercise acceptable, citing advantages such as independence from time, location, and climate.

P3: "It is preferable to exercise using the TikTok platform because time is more flexible, and you can exercise whenever you want. At the same time, it is not venue-specific and can be done at home or in the community. It is also unaffected by the weather; I can exercise at home whether it is excessively chilly or hot."
P9: "The most significant advantage of TikTok is its ability to move freely. If you establish a fixed time and place, it may be impossible to complete because everyone's activity time is different. TikTok is excellent, which makes it possible for us to exercise together when we have free time. We also can exercise at home when the weather is terrible, or the schedule is unpredictable."
P12: "The TikTok platform provides an excellent platform to exercise. The best aspect is that I'm not restricted by time. I exercise in the mornings, afternoons, and evenings. I am not like other people who do fixed exercise in the morning. I am quite casual, and I only exercise when I really want. I do not always force myself to exercise. So, the way using TikTok makes me feel free. Another advantage is that the location is also free. I saw them go out to square dance, which I dislike. I prefer to stay at home since it is more comfortable. When the weather is nice, I take my phone holder to the park and exercise. When the weather is poor, I exercise at home."
P27: "TikTok platform is extremely good and convenient for exercise. One of the advantages is that it's freer and I do exercise whenever I have time. My exercise time is not fixed, and I usually adjust it. Sometimes with other participants, and sometimes alone at home, both are flexible and not rigid."

3) Behaviour, physical and mental changes

All participants experienced varying degrees of advantageous alterations in their subjective feelings, encompassing both behaviour, physical, and psychological aspects. Following the exercise intervention, 25 (96.15%) participants reported enhanced strength in their hands and feet. 18 (69.23%) noted an improved mood. 17 (65.38%) experienced greater overall relaxation on the whole body. 16 (61.54%) indicated improved sleep quality. 12 (46.15%) altered their diet, including increased protein consumption. Eight (30.77%) reported an enhanced appetite. Five (19.23%) indicated an enhancement in gastrointestinal function, including alleviation of constipation. Four (15.38%) showed increased energy level when doing chores in their daily life. In addition, all participants expressed their willingness to learn more about sarcopenia after finishing this project, and to persist in exercising to prevent sarcopenia, and to disseminate information regarding sarcopenia to family and friends.

P5: "My physique has improved after enrolling in your program. I used to have weak walking feet, but after completing this training, I felt stronger in my hands and feet. Originally, my physical condition was general; now, I feel much better. I also have a

pleasant spirit and become energetic every day. Sleep has also improved. I now go to bed around nine or ten p.m. and get up around five or six. My gastrointestinal function was slightly constipated before, and it took three or four days to poop once, but now I poop virtually every day, and my bowel movements have been more open. My diet used to appear like there was nothing to eat, but now my appetite has improved, and I've gained 10 pounds. My mood is excellent because my body is becoming healthy."

P12: "Following exercise, I notice a significant difference. After practicing your exercise, my entire body feels lighter. I hadn't exercised much previously, and I became fatigued rapidly with whatever I did. My first impression after exercise following your video's is that my entire body became relaxed. I seem to be able to handle chores and care for grandchildren with greater vigour and a better spirit. There's also changes in my hands, which used to be weak but has suddenly grown significantly stronger now. Besides, I feel considerably better in my mood. In the past, if I stayed in one area for an extended period, I would become melancholy and upset when cleaning and other tasks. The noticeable change after doing this exercise is that the body is relaxed, then my mood is improved, and the strength to perform things is increased, and there is a lot of happiness. Sleep, which used to last until two or three a.m., always wake up in the middle of the night. Many older people suffer from this, and our sleep quality is poor. After completing this exercise, I usually fall asleep around eleven o'clock and can sleep until five or six a.m. next day and can sleep all night. In terms of food, exercise improves my gastrointestinal function. Initially, I had some constipation, but after finishing the exercise, my bowel movements have become more open. I used to have bowel movements every few days, but now I have them every day or two. My appetite has improved slightly now that my bowel movement is no longer restricted. Originally, it turned out that I ate at home with my children, and I didn't pay attention to how to eat. I usually ate whatever I wanted, and didn't pay attention to protein intake. After completing this project, when I cook, I will consider meat, like pig and beef, and choose something with more lean meat and less fat. Because our family consumes a lot of fat meat, as I believe that fat meat is more delicious to eat, but I will pay more attention to purchasing lean meat now. It turned out that I don't like eating eggs; instead, I eat steamed bread noodles in the morning, and then, as you suggested, I'll insist on eating an egg every day. This information has assisted me in cooking, purchasing food, and experimenting with different dietary combinations.

I was previously unaware of sarcopenia, but now that I have been aware of it through your project. I will consciously pay attention to sarcopenia information. I would like to continue using your exercise strategy because it is so convenient, and I want to keep with it. I am also prepared to acquire and maintain an exercise habit to avoid poor health, which is beneficial to me. I am willing to provide necessary information

about sarcopenia to my family and friends. I've asked you before if you could send it to my family and friends so we could view it together. Many older people in my family do not exercise. However, you currently don't enable video sharing, right? Just see if you have any videos that we could share with our relatives and friends."
P17: "My hands feel stronger now after undertaking this exercise. There are also other changes, and I feel a little lighter in my whole body than previously. I used to have occasional problems with my nerves because I had cerebral arteriosclerosis, and I couldn't stretch my limbs, which were rigid. After completing this exercise, my body and limbs are stretched out. As for eating, I notice that my stomach performs considerably better now. I used to suffer from atrophic gastritis, which caused some discomfort or distress. I used to dislike eggs and milk, but after coming to your project and watching those health education videos, I started insisting on an egg and a cup of hot milk every day. Now that my stomach function has improved. In terms of sleep, I normally fall asleep late, or wake up in the middle night, but my sleep has improved since I started exercising. Now I understand the benefit of exercise; if I don't exercise for two days, I feel uncomfortable, so I will insist on exercise.
I am eager to learn more about sarcopenia and continue insisting on this exercise. I'll also get into the habit of exercising since now that I'm used to it. It's more uncomfortable without exercise in my life now. Before participating in this project, I took a stroll after every meal and sit still at home. After engaging in this research, I discovered that walking only exercises my feet, not my hands, therefore I exercised my hands by lifting water bottles. I take videos of my exercises and share them to my two sisters. My elder sister is also in her sixties, and he is currently doing exercise with me. Every day, we upload exercise footage to our Wechat group and share information with one another."

· ***Theme 3: The recommendations for future promotion***

Ten participants identified obstacles and dilemmas regarding future promotion of this study. For instance, there are barriers in the promotion to older adults lacking smartphones, possessing diminished cognitive capacity, and exhibiting low health literacy. Only three participants proposed enhancements, such as creating a hybrid of online and offline formats and enhancing early-stage publicity.

P5: "Some senior people, such as those without smart phones, those suffering from Alzheimer's disease, and those with disabled hands and feet, may be unable to participate in the study. People with outdated notions, like who do not want to exercise and just play mahjong, may not be highly involved."
P12: "Older people without smartphones may not be able to participate. For example, I have a sister who does not have a mobile phone, so if she wants to

participate, it will have great difficulties. Maybe I wonder if you can combine, for example, some people come to organize activities in the community, I can help introduce to my friends and family. If we have a mobile phone, we can do it ourselves on TikTok. So I think it might be a little difficult not to have a smart phone."

P25: "I estimate that three or four out of ten people would be willing to attend. Our older population has a low level of knowledge, and most individuals are unfamiliar with sarcopenia. Without this concept, it will be unappreciated. In my opinion, the most significant way to promote this idea is to generate publicity and popularity. Professionals must talk clearly so that older people can pay attention, and only then will they do so. You can locate professionals to start more classes in the community so that older population can better comprehend and promote this disease."

P28: "Some senior individuals may experience various physical ailments as they age, including falls, dementia, and diminished reaction times, which may hinder their ability to participate. Nevertheless, the young-old individuals should be deemed acceptable."

P32: "In terms of promotion, I suppose if the person truly has subjective awareness to protect health or wishes to take their body more seriously, he might participate. Some older people are stubborn, and they think it doesn't matter with their health. They believe their bodies are great, and then lazy to move, for a variety of reasons. It really depends on individuals. Besides, publicity is quite crucial, because the old, the level of education is not very high, so they do not know much about this disease. This project will indeed improve our health, so to strengthen publicity in the early stages is necessary."

CONSORT 2010 checklist of information to include when reporting a pilot or feasibility trial*

Section/Topic	Item No	Checklist item	Reported on page No
**Title and abstract**			
	1a	Identification as a pilot or feasibility randomised trial in the title	P1-2
	1b	Structured summary of pilot trial design, methods, results, and conclusions (for specific guidance see CONSORT abstract extension for pilot trials)	P2
**Introduction**			
Background and objectives	2a	Scientific background and explanation of rationale for future definitive trial, and reasons for randomised pilot trial	P3-5
	2b	Specific objectives or research questions for pilot trial	P4-5
**Methods**			
Trial design	3a	Description of pilot trial design (such as parallel, factorial) including allocation ratio	P19-20
	3b	Important changes to methods after pilot trial commencement (such as eligibility criteria), with reasons	N/A
Participants	4a	Eligibility criteria for participants	P20
	4b	Settings and locations where the data were collected	P20
	4c	How participants were identified and consented	P21-22
Interventions	5	The interventions for each group with sufficient details to allow replication, including how and when they were actually administered	P22
Outcomes	6a	Completely defined prespecified assessments or measurements to address each pilot trial objective specified in 2b, including how and when they were assessed	P22-24
	6b	Any changes to pilot trial assessments or measurements after the pilot trial commenced, with reasons	N/A
	6c	If applicable, prespecified criteria used to judge whether, or how, to proceed with future definitive trial	N/A
Sample size	7a	Rationale for numbers in the pilot trial	P20-21
	7b	When applicable, explanation of any interim analyses and stopping guidelines	N/A
Randomisation:			
Sequence generation	8a	Method used to generate the random allocation sequence	N/A
	8b	Type of randomisation(s); details of any restriction (such as blocking and block size)	N/A
Allocation concealment mechanism	9	Mechanism used to implement the random allocation sequence (such as sequentially numbered containers), describing any steps taken to conceal the sequence until interventions were assigned	N/A

Implementation	10	Who generated the random allocation sequence, who enrolled participants, and who assigned participants to interventions	N/A
Blinding	11a	If done, who was blinded after assignment to interventions (for example, participants, care providers, those assessing outcomes) and how	N/A
	11b	If relevant, description of the similarity of interventions	N/A
Statistical methods	12	Methods used to address each pilot trial objective whether qualitative or quantitative	P24-25
**Results**			
Participant flow (a diagram is strongly recommended)	13a	For each group, the numbers of participants who were approached and/or assessed for eligibility, randomly assigned, received intended treatment, and were assessed for each objective	P5
	13b	For each group, losses and exclusions after randomisation, together with reasons	P5
Recruitment	14a	Dates defining the periods of recruitment and follow-up	P5-6
	14b	Why the pilot trial ended or was stopped	N/A
Baseline data	15	A table showing baseline demographic and clinical characteristics for each group	P6
Numbers analysed	16	For each objective, number of participants (denominator) included in each analysis. If relevant, these numbers should be by randomised group	P6
Outcomes and estimation	17	For each objective, results including expressions of uncertainty (such as 95% confidence interval) for any estimates. If relevant, these results should be by randomised group	P7-12
Ancillary analyses	18	Results of any other analyses performed that could be used to inform the future definitive trial	P8-10
Harms	19	All important harms or unintended effects in each group (for specific guidance see CONSORT for harms)	P6-7
	19a	If relevant, other important unintended consequences	N/A
**Discussion**			
Limitations	20	Pilot trial limitations, addressing sources of potential bias and remaining uncertainty about feasibility	P18
Generalisability	21	Generalisability (applicability) of pilot trial methods and findings to future definitive trial and other studies	P18-19
Interpretation	22	Interpretation consistent with pilot trial objectives and findings, balancing potential benefits and harms, and considering other relevant evidence	P13-17
	22a	Implications for progression from pilot to future definitive trial, including any proposed amendments	P13-19
**Other information**			
Registration	23	Registration number for pilot trial and name of trial registry	P19-20
Protocol	24	Where the pilot trial protocol can be accessed, if available	P19-20
Funding	25	Sources of funding and other support (such as supply of drugs), role of funders	P26
	26	Ethical approval or approval by research review committee, confirmed with reference number	P20

Citation: Eldridge SM, Chan CL, Campbell MJ, Bond CM, Hopewell S, Thabane L, et al. CONSORT 2010 statement: extension to randomised pilot and feasibility trials. BMJ. 2016;355. This is an Open Access article distributed in accordance with the terms of the Creative Commons Attribution (CC BY 3.0) license (http://creativecommons.org/licenses/by/3.0/), which permits others to distribute, remix, adapt and build upon this work, for commercial use, provided the original work is properly cited.

*We strongly recommend reading this statement in conjunction with the CONSORT 2010, extension to randomised pilot and feasibility trials, Explanation and Elaboration for important clarifications on all the items. If relevant, we also recommend reading CONSORT extensions for cluster randomised trials, non-inferiority and equivalence trials, non-pharmacological treatments, herbal interventions, and pragmatic trials. Additional extensions are forthcoming: for those and for up-to-date references relevant to this checklist, see www.consort-statement.org.
